# Structural and Functional Characterization of the VQ Protein Family and VQ Protein Variants from Soybean

**DOI:** 10.1038/srep34663

**Published:** 2016-10-06

**Authors:** Yuan Zhou, Yan Yang, Xinjian Zhou, Yingjun Chi, Baofang Fan, Zhixiang Chen

**Affiliations:** 1 Department of Horticulture, Zijingang Campus, 866 Yuhangtang Road, Zhejiang University, Hangzhou, 310058, China; 2 Department of Botany and Plant Pathology, 915 W. State Street, Purdue University, West Lafayette, IN 47907, USA

## Abstract

Proteins containing the FxxxVQxhTG or VQ motif interact with WRKY transcription factors. Although VQ proteins have been reported in several plants, knowledge about their structures, functions and evolution is still very limited. Here, we report structural and functional analysis of the VQ protein family from soybean. Like Arabidopsis homologues, soybean VQ proteins bind only Group I and IIc WRKY proteins and a substantial number of their genes are responsive to stress-associated phytohormones. Overexpression of some soybean VQ genes in Arabidopsis had strong effects on plant growth, development, disease resistance and heat tolerance. Phylogenetic analysis, sequence alignment and site-directed mutagenesis revealed that the region immediately upstream of the FxxxVQxhTG motif also affects binding to WRKY proteins. Consistent with a larger WRKY-binding VQ domain, soybean VQ22 protein from cultivated soybean contains a 4-amino acid deletion in the region preceding its VQ motif that completely abolishes its binding to WRKY proteins. By contrast, the 4-amino acid deletion is absent in the VQ22 protein from wild soybean species (*Glycine soja*). Overexpression of wild soybean *VQ22* in cultivated soybean inhibited growth, particularly after cold treatment. Thus, the mutation of soybean VQ22 is associated with advantageous phenotypes and may have been positively selected during evolution.

As sessile organisms, plants are constantly exposed to a wide range of environmental conditions, favorable or unfavorable, and rely on complex molecular, biochemical and cellular processes to adapt or survive. Differential gene transcription through concerted action of transcription factors is central to the adaptive processes of plants. While many families of plant transcription factors have homologs in other organisms, some are plant-specific and, therefore, may act as regulators of biological processes unique to plants. WRKY proteins are a large superfamily of sequence-specific DNA-binding transcription factors found almost exclusively in plants^1^. WRKY proteins contain the highly conserved WRKY domain, which contain the almost invariant WRKYGQK sequence at the N-terminus followed by a C2H2 or C2HC zinc-finger motif^1^. According to the number and sequence of the conserved WRKY zinc-finger motifs, WRKY proteins were initially classified into three groups^1^. The first group contains two C2H2 zinc-finger motifs, whereas the second group contains one C2H2 zinc-finger motif, and the third group contains one C2HC zinc-finger motif. More recent analyses have shown that Group II WRKY proteins can be further divided into five subgroups (IIa, IIb, IIc, IId, and IIe)^1^,^2^. Since their initial identification more than 20 years ago, plant WRKY transcription factors have been subjected to extensive analysis for their biological functions. These studies have established plant WRKY transcription factors as critical regulators of plant responses to a wide spectrum of biotic and abiotic stresses^3^,^4^,^5^,^6^,^7^,^8^,^9^,^10^,^11^. Some plant WRKY proteins are also involved in the regulation of plant growth and development such as trichome^12^ and seed development^13^, germination^14^ and senescence^15^,^16^. Therefore, plant WRKY transcription factors are important regulators of diverse biological processes in plants.

Like many other regulatory proteins, WRKY transcription factors rarely act alone but rather often function as complexes with their interacting partners for regulation of important properties such as DNA-binding and transcription-regulating activities, stabilities and subcellular localization^17^. Studies over the last decade have identified a substantial number of WRKY-interacting proteins with roles in signaling, transcription, chromatin remodeling and other cellular processes^17^. Among these WRKY-interacting partners are proteins containing a conserved FxxxVQxLTG or VQ motif^18^. VQ proteins are encoded by a fairly large family with 25, 34 and 39 members in *Physcomitrella patens*, Arabidopsis and rice, respectively^19^,^20^. Using yeast two-hybrid assays, we have previously shown that Arabidopsis VQ proteins specifically interact with the C-terminal WRKY domain of Group I WRKY proteins and the single WRKY domain of Group IIc WRKY proteins^18^. A number of VQ proteins have also been subjected to molecular and genetic analysis for their biological functions. We have previously discovered that two VQ proteins from *Arabidopsis thaliana*, SIB1(AtVQ23) and SIB2 (AtVQ16), act as co-activators of AtWRKY33 in plant defense against necrotrophic pathogens by specifically recognizing the C-terminal WRKY domain and stimulating the DNA-binding activity of WRKY33^10^. Intriguingly, a number of other Arabidopsis VQ proteins including AtVQ21/MKS1, AtVQ22, AtVQ12 and AtVQ29 function as negative regulators of plant defense against necrotrophic pathogens^21^,^22^,^23^,^24^. The opposite roles of different VQ proteins in plant defense suggest the complex nature of plant defense involving interacting pathways that require tight regulation and modulation not only for effective defense against different types of microbial pathogens but also for balancing defense with growth. Other VQ proteins regulate plant responses to abiotic stress. AtVQ15 is a Calmodulin-binding protein and acts as a negative regulator in response to osmotic stress^25^. AtVQ9 is a AtWRKY8-interacting protein but its mutation has an opposite effect that that of AtWRKY8 on salt tolerance, suggesting that the VQ protein may function as a transcriptional repressor that antagonizes AtWRKY8 during plant response to salt stress^26^. A number of plant VQ proteins also regulate plant growth and development. Arabidopsis AtVQ14/IKU1 and its interacting WRKY10/MINI3 are both important regulators of endosperm development and seed size^27^. AtVQ29 negatively regulates seedling photomorphogenesis^28^ whereas AtVQ8 may regulate chloroplast development^18^. The molecular and genetic analysis of these Arabidopsis VQ genes indicates their important roles in diverse biological processes in plants.

Although significant progresses have been made in the studies of VQ proteins, our understanding of this important family of transcription regulator is still very limited. Gene encoding VQ proteins have been identified in crop plants including rice^20^, Chinese cabbage^29^ and grapevine^30^ in which the interactions with VviWRKYs were predicted^31^, but there have been no molecular and genetic studies that rigorously interrogate the biological functions of the VQ genes in crop plants. Even in Arabidopsis, there have been only a few studies that provide in-depth analysis of the structures and functions of its VQ genes and gene products. As a result, there remain important gaps in our knowledge about the structure-function relationship and the evolution of plant VQ proteins. In the present study, we have analyzed the entire family of 74 structurally divergent VQ proteins from soybean. We demonstrate that soybean VQ proteins bind only Group I and Group IIc WRKY proteins but they differ greatly in binding specificity. Using phylogenetic analysis, sequence alignment and site-directed mutagenesis, we further revealed that the region immediately upstream of the short FxxxVQxhTG motifs also affects binding to WRKY proteins, thereby expanding the functional domain of VQ proteins for binding to WRKY proteins. Through genome-wide analysis, we also identified a VQ variant in cultivated soybean with a 4-amino acid deletion in the region preceding its FxxxVQxhTG motif that completely abolishes its activity to bind WRKY proteins. The 4-amino acid deletion apparently occurred recently as it is absent in the VQ protein from wild soybean species (*G. soja*). Molecular genetic analysis suggests that the loss-of-function mutation of the soybean VQ protein may have been positively selected during evolution because it is associated with advantageous phenotypes in growth.

## Results

### Identification and structural analysis of soybean VQ protein family

**Figure 1:**
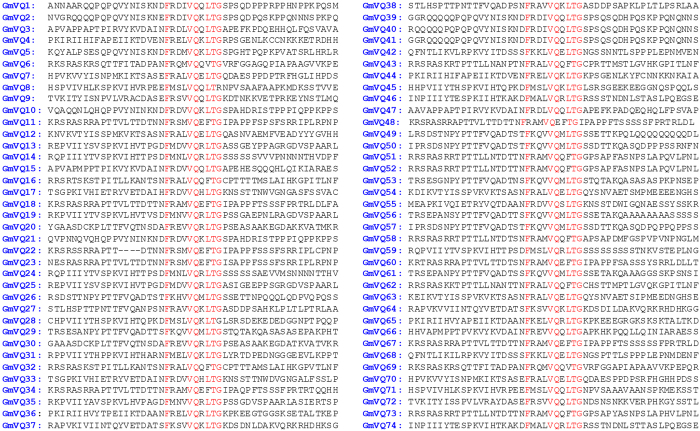
VQ domain sequence of soybean VQ proteins. The highly conserved residues in the FxxxVQxhTG motif are shown in red.

**Figure 2:**
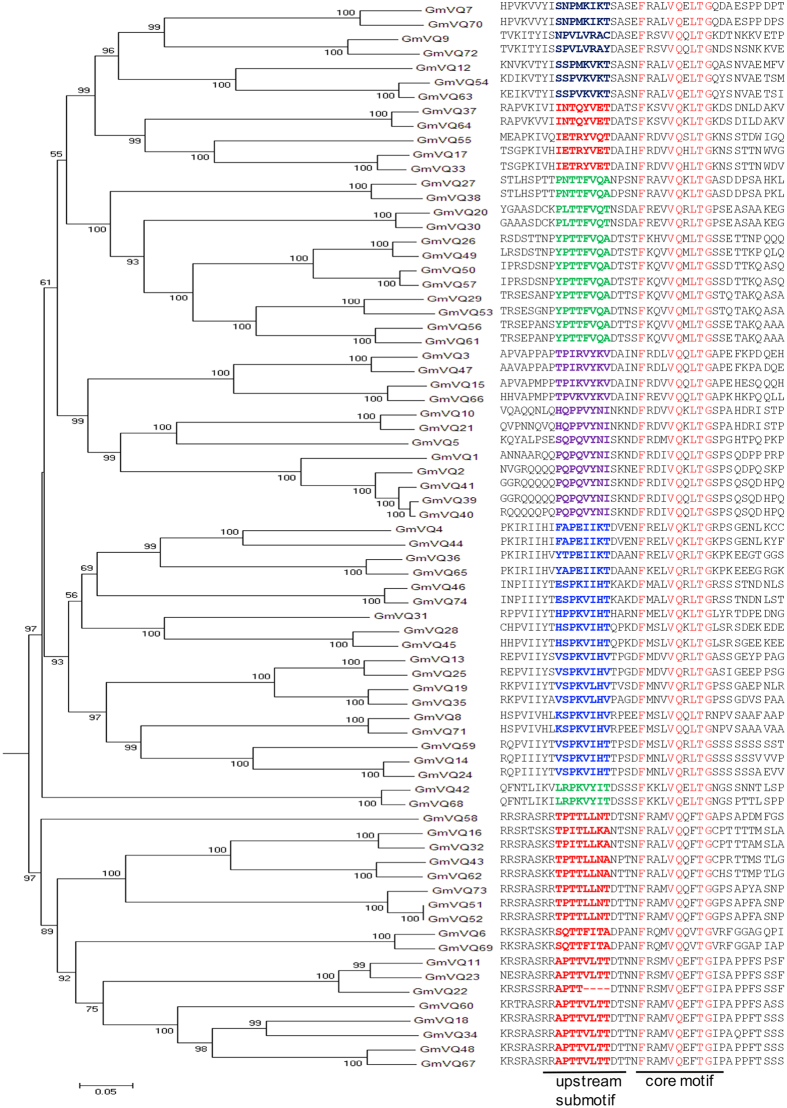
Phylogenetic analysis of soybean VQ proteins. The phylogenetic tree was inferred using the the neighbor-joining method from 74 soybean VQ proteins. Bootstrap values from 1,000 replicates were used to assess the robustness of the tree. VQ domain sequences are also shown for each VQ protein. The highly conserved residues in the FxxxVQxhTG motif are in red. Amino acid residues of the upstream submotifs are shown in different colors for different clades.

Identification of the soybean VQ gene family with 74 members through searching the Phytozome Database has recently been reported^32^. However, in Table 1 of the reported study, the names and Phytozome Identifiers (IDs) of a majority of the 74 soybean VQ proteins were mismatched with their corresponding VQ motif sequences (Supplemented Table 1). As a result, it is unclear whether one can match individually the reported structural and expression properties with the 74 soybean VQ genes and their protein products. Using the same database, we also identified 74 genes encoding proteins containing the FxxxVQxxhTG motif in soybean (Fig. 1). As found in Arabidopsis, a majority of soybean VQ genes are also intronless and encode relatively small proteins with fewer than 300 aa residues. Thus, the VQ gene family from soybean is substantially large from those of other plants such as Arabidopsis (34 members) and rice (39 members). This is likely due to the fact that soybean is a paleopolyploid that has undergone at least two rounds of large-scale duplication at approximately 14 and 42 million years ago^33^. Indeed, alignment of amino acid residues and phylogenetic tree construction revealed that a majority of the soybean VQ proteins have one or more close homologs (Fig. 2). Among these close VQ homologs, some (e.g. GmVQ22/GmVQ23 and GmVQ39/GmVQ40/GmVQ41) are tandem repeats. GmVQ36 and GmVQ37 on chromosome IIX are closely related to GmVQ65 and GmVQ64 on chromosome XV, respectively and the synonymous distances between the two gene pairs are very similar (Fig. 2). Likewise, GmVQ16, GmVQ17, GmVQ18 and GmVQ19 on chromosome V are close homologs of GmVQ32, GmVQ33, GmVQ34 and GmVQ35 on chromosome IIX, respectively and the distances between the four gene pairs are also very similar. These observations suggest that a large number of close VQ homologs in soybean resulted from duplication of chromosome regions or even whole chromosomes (polyploids).

All 74 soybean VQ proteins contain the conserved FxxxVQxxhTG motif. Among the 74 VQ protein, 56 contain the FxxxVQxxLTG motif, 16 contain FxxxVQxxFTG motif and 2 contain the FxxxVQxxVTG motif (Figs 1 and 2). All these slightly varied VQ motifs are also found in VQ proteins from Arabidopsis^18^, indicating that they are conserved across plant species. A notable discovery from the sequence alignment of the VQ motifs is a 4-amino acid deletion in GmVQ22 in a region immediately upstream of the conserved FxxxVQxxFTG motif (Figs 1 and 2). As will be described later, deletion of the 4 amino acid residues in GmVQ22 affects its binding to WRKY proteins, suggesting that the functional VQ domains for binding of WRKY proteins may be larger than the highly conserved FxxxVQxxFTG motif. The phylogenetic analysis of the 74 soybean VQ proteins is consistent with this possibility. As shown in Fig. 2, the phylogenetic tree consists of a number of clades, which provide further information about the structural relationship among the VQ proteins. First, we observed that the soybean VQ proteins with FxxxVQxxLTG motif are classified into one branch of closely related clades, whereas those VQ proteins with the FxxxVQxxFTG or FxxxVQxxVTG are grouped into the other branch of clades (Fig. 2). This observation supports that the FxxxVQxxhTG motif is the most important determinant of the phylogenetic relationship of the VQ protein family. Second, within both branches of the phylogenetic tree, the amino acid sequences corresponding to those deleted in GmVQ22 can be divided into several types that are largely clade-specific, suggesting that subfamilies of VQ proteins are determined largely by the sequences immediately upstream of the conserved FxxxVQxxhTG motif (Fig. 2). Third, in the second branch of the phylogenetic tree, 14 VQ proteins with the FxxxVQxxFTG motif contain a TTL/VL upstream submit, whereas two VQ proteins containing the FxxxVQxxVTG motif have a TTFI upstream submotif (Fig. 2). Thus, the loss of an aromatic amino acid in the FxxxVQxxVTG motif of GmVQ6 and GmVQ60 appears to be associated with the complementary addition of an aromatic acid residue in the upstream submotif (Fig. 2).

### Interacting WRKY partners of GmVQ proteins

**Figure 3:**
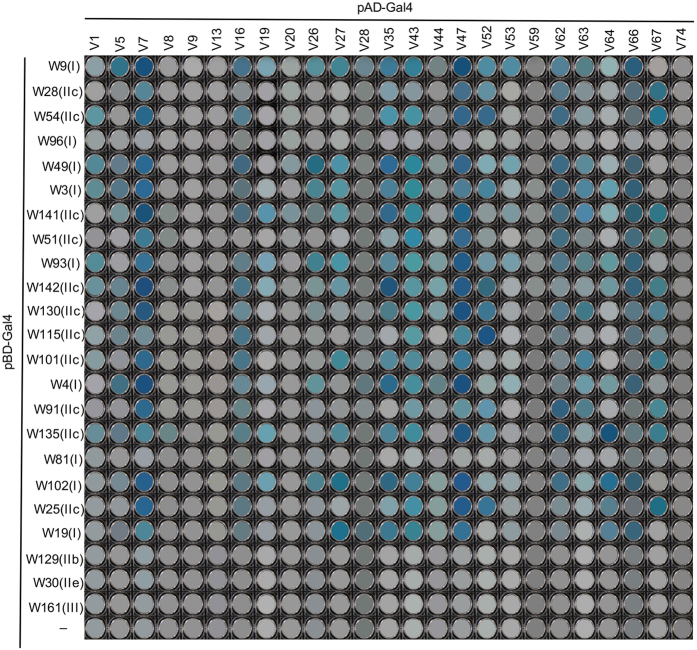
Interaction of GmVQ proteins with GmWRKY proteins in yeast cells. The Gal4 DNA BD-WRKY (W) domain fusion bait vectors were co-transformed with the activation domain (AD)-VQ (V) fusion prey vectors into yeast cells and the transformant cells were assayed for *LacZ* reporter gene expression. The empty pBD prey vector was used as negative control. The specific subfamilies to which the tested WRKY proteins belong to are indicated in parentheses.

Arabidopsis VQ proteins interact specifically with the C-terminal WRKY domain of Group I WRKY proteins and the single WRKY domain of Group IIc WRKY proteins^18^. Using yeast two-hybrid (Y2H) assays, we examined the partnership between soybean WRKY and VQ proteins. We selected 25 soybean VQ proteins from different clades of the phylogenetic tree and fused their corresponding genes with the activation domain (AD) of the Gal4 transcription factor in the Y2H prey vector pAD-Gal4 (Fig. 3). We also fused the DNA fragments for the C-terminal WRKY domain of 9 soybean Group I WRKY proteins and the single WRKY domain of 11 Group IIc WRKY proteins with the DNA-binding domain (BD) of the Gal4 transcription factor in the Y2H bait vector pBD-Gal4 (Fig. 3). As negative controls, the DNA fragments for the WRKY domains of three Group IIb, IIe and III WRKY proteins were also cloned into pBD-Gal4 (Fig. 3). The fused pAD and pBD recombinant vectors were then co-transformed into yeast cells and tested for *LacZ* reporter gene expression through assays of β-galactosidase activity. As shown in Fig. 3, 21 of the 25 soybean VQ proteins tested interacted with some of the Group I and Group IIc WRKY proteins, albeit with varying intensities based on the β-galactosidase activity. By contrast, none of the 25 VQ proteins interacted with the WRKY domains of Group IIb, IIe or III WRKY proteins (Fig. 3). These results indicated that like Arabidopsis homologs, soybean VQ proteins also appear to interact only with the C-terminal WRKY domain of Group I WRKY proteins and the single WRKY domain of the Group IIc WRKY proteins. Among the 25 tested soybean VQ proteins, some including GmVQ7, GmVQ16, GmVQ47 and GmVQ62 and GmVQ66 interacted with almost all 20 tested Group I and Group IIc WRKY proteins (Fig. 3). A majority of the other tested soybean VQ proteins, however, interacted only with a selected number of Group I and Group IIc WRKY proteins (Fig. 3). A few VQ proteins including GmVQ8, GmVQ20 and GmVQ28, on the other hand, interacted with only a very few Group I or Group IIc WRKY proteins (Fig. 3). Four tested VQ proteins (GmVQ9, GmVQ13, GmVQ59 and GmVQ74) failed to interact with any of the 20 tested Group I or Group IIc WRKY proteins (Fig. 3). Thus, soybean VQ proteins display a substantial diversity in their binding specificity to WRKY proteins.

### Expression analysis of soybean VQ genes

**Figure 4:**
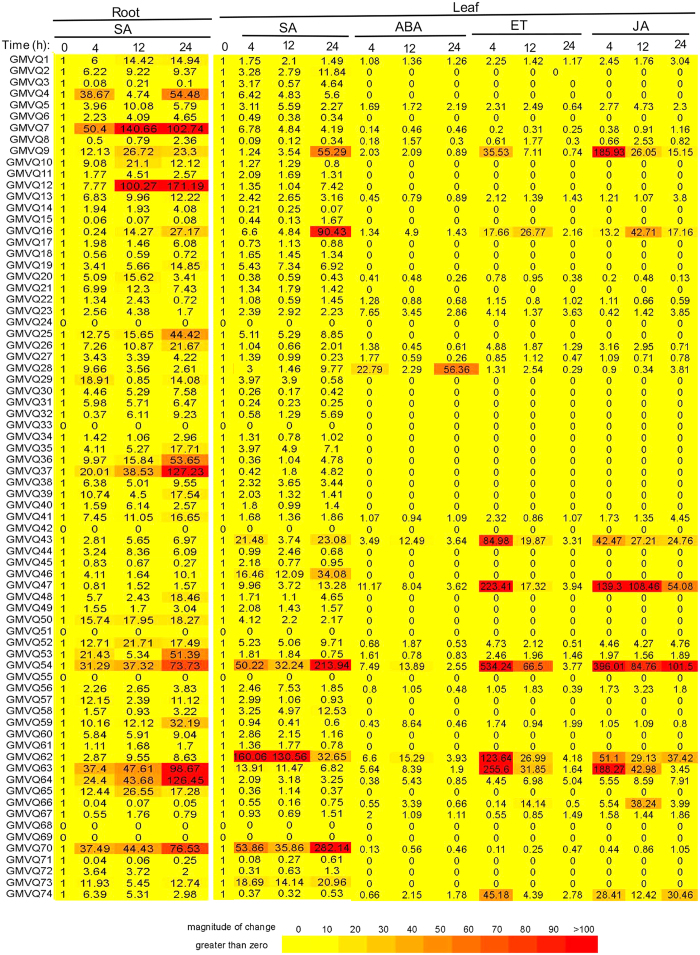
Expression of soybean GmVQ genes in response to different hormones. Roots of 2-week old soybean seedlings (*G. max* cv ‘Williams 82’) were treated with 1 mM SA. The first trifoliolate leaves of about 2-week-old soybean seedlings were treated with 0.1 mM ABA, 0.5 mM ET and JA and 1 mM SA. Plant tissues were collected from three indeoednent biologcal repeats at indicated time of treatment with the phytohormones for RNA isolation and qRT-PCR analysis of the VQ gene transcripts using gene-specific primers. The numbers in the expression profile are from three technical repeats and are normalized induction fold over untreated control plants. Those VQ genes that were not analyzed for a specific phytohormone were given a number of zero.

In Arabidopsis, a large percentage of WRKY and VQ genes are responsive to biotic and abiotic stresses, supporting that the predominant roles of the two gene families in plant stress responses^4^,^18^. Induction of plant stress responses is associated with signaling of a number of phytohormones including salicylic acid (SA), abscisic acid (ABA), jasmonic acid (JA) and ethylene (ET). To investigate possible roles of soybean VQ genes in plant stress responses, therefore, we examined their expression in response to SA, ABA, ET and JA using quantitative real-time PCR (qRT-PCR). First, we analyzed in both root and leaf tissues the SA-responsiveness of the 67 soybean VQ genes that were initially identified based on the Wm82.a1.v1 annotation version of the soybean genome. In roots, 58 out of the 67 VQ genes examined displayed >2-fold induction at least at one of the three time points after SA treatment (Fig. 4), indicating that a vast majority of soybean VQ genes are responsive to SA in roots. In leave, 40 out of the 67 analyzed GmVQ genes (~60%) displayed >2-fold induction at least at one of the three time points after SA treatment (Fig. 4). We also analyzed about 30 soybean VQ genes selected from all clades of the phylogenetic tree for responses to ABA, ET and JA and observed that approximately 20 of them were induced by >2-fold by at least one of the three phytohormones (Fig. 4). Interestingly, a number of soybean VQ genes including *GmVQ9*, *GmVQ16*, *GmVQ43*, *GmVQ47*, *GmVQ54*, *GmVQ63* and *GmVQ64* were induced at high levels by all four phytohormones. The high responsiveness of these VQ genes to distinct types of stress-related phytohormones suggest their broad roles in plant stress responses.

### Functional analysis of soybean VQ genes in Arabidopsis

**Figure 5:**
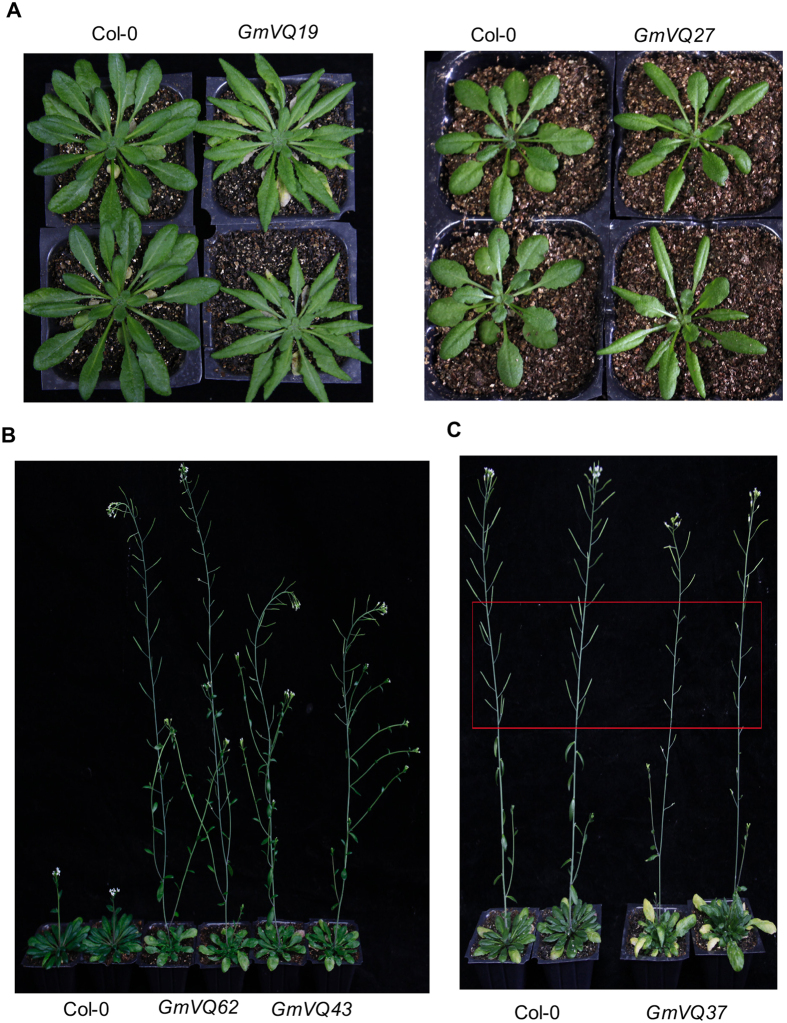
Growth and developmental phenotypes of transgenic VQ-overexpressing *Arabidopsis* plants. (**A**) Altered leaf growth of transgenic plants overexpressing Gm*VQ19* and *GmVQ27*. The picture of Col-0 wild type and two lines of transgenic overexpression plants for each VQ gene was taken 7 weeks after germination. (**B**) Earlier flowering of transgenic plants overexpressing Gm*VQ62* and *GmVQ43*. The picture of Col-0 wild type and two lines of transgenic overexpression plants for *GmVQ* gene was taken 10 weeks after germination. (**C**) Reduced seed setting of transgenic plants overexpressing *GmVQ37*. The picture of Col-0 wild type and two lines of transgenic plants was taken 12 weeks after germination. The parts of the inflorescences of the transgenic plants with poor silique development and seed setting are indicated by a red box.

As an approach for functional analysis of the soybean *VQ* genes, we generated transgenic Arabidopsis plants that constitutively overexpressed soybean *VQ* genes. The coding sequences of soybean Gm*VQ* genes including those highly responsive to stress-related phytohormones were subcloned behind the *CaMV 35S* promoter in a binary plant transformation vector and transformed into *Arabidopsis* plants. qRT-PCR was used to identify F0 transformant plants with elevated levels of transcripts for the transformed VQ gene. Due to the large number of transgenic lines for a large number of soybean VQ genes, our analysis of the transgenic plants was carried out in transgenic F1 lines with increased VQ gene expression and focused primarily on alterations in growth, development and disease resistance. Generally speaking, overexpression of a majority of the soybean VQ genes had no effects on growth or development of transgenic plants. However, altered growth was observed in transgenic plants overexpressing some of the soybean VQ genes. For example, transgenic plants with elevated transcript levels for Gm*VQ19* or Gm*VQ27* had narrower leaves than those of untransformed control plants (Fig. 5A). Transgenic plants overexpressing Gm*VQ43 and GmVQ62* flowered substantially earlier than control plants (Fig. 5B). Overexpression of GmVQ37 had no significant effect on growth and flowering time but reduced seed setting, particularly on the first 10 or so siliques (Fig. 5C). The reduced seed setting appeared to be associated with shorter filaments of the stamen in transgenic plant flowers, which would reduce pollination.

**Figure 6:**
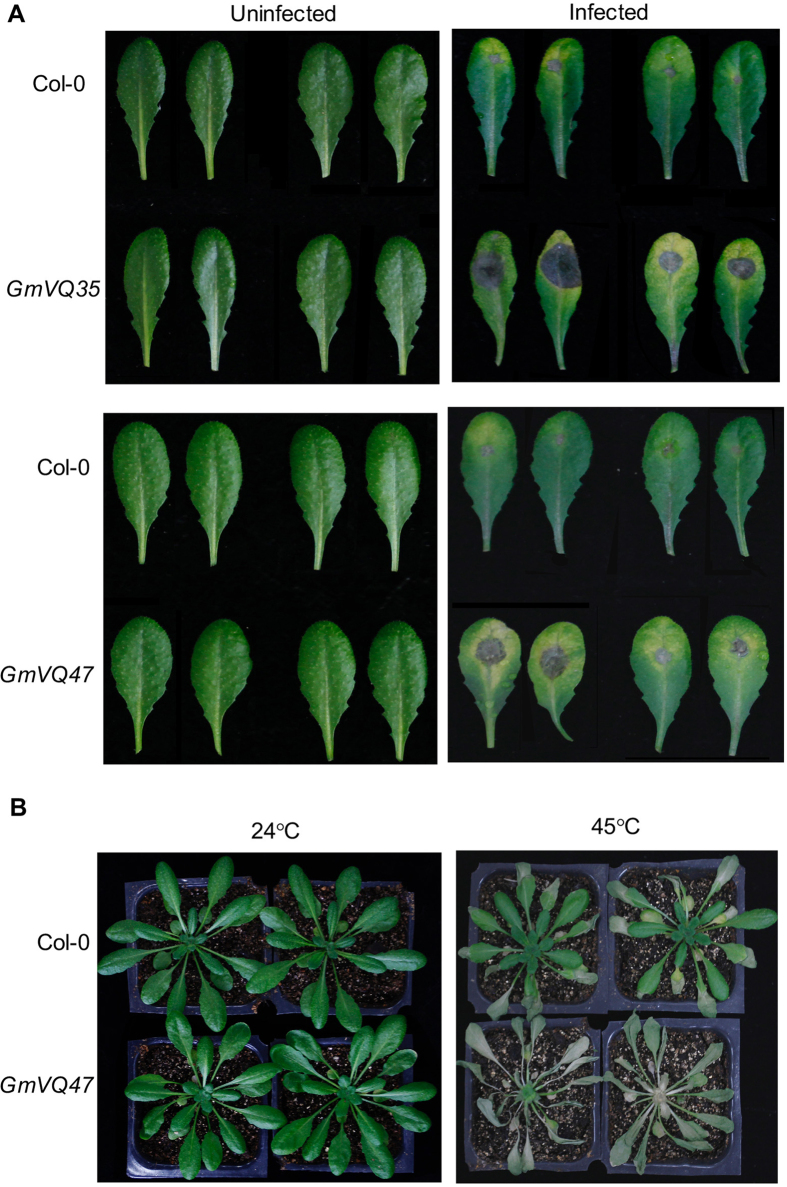
Altered disease resistance and heat tolerance of transgenic Gm*VQ*-overexpressing *Arabidopsis* plants. (**A**) Enhanced susceptibility to *B. cinerea*. Fully expanded leaves of Col-0 wild type and two independent lines of transgenic plants expressing Gm*VQ35* or *GmVQ47* were drop-inoculated with *Botrytis* and the picture was taken at the 4th day post inoculation (dpi). Both uninoculated and inoculated leaves are shown (**B**). Enhanced heat sensitivity. Col-0 wild type and transgenic plants expressing *GmVQ47* were placed in growth chamber 45 °C for 10 hours. The plants were then moved back to a growth room at 25 °C and pictures of representative plants were taken after 4-day recovery.

A number of Arabidopsis VQ proteins have been show to play roles in plant resistance to necrotrophic pathogens^21^,^22^,^23^,^24^. Therefore, we examined effects of overexpression of soybean VQ genes on response of transgenic Arabidopsis lines to *B. cinerea*. Using drop-inoculation assays of detached leaves, we observed that the lesion sizes of transgenic lines overexpressing *GmVQ35* and *GmVQ47* were substantially larger than those of control wild-type plants (Fig. 6A), suggesting that the two soybean VQ proteins act as negative regulators of plant defense against the necrotrophic pathogen. Transgenic Arabidopsis plants overexpressing *GmVQ47* also displayed increased sensitivity to heat (Fig. 6B). Thus, expression of a number of soybean VQ genes had various effects on plant growth, development and responses to both biotic and abiotic stresses.

### Identification of VQ22 allelic variants in soybean

**Figure 7:**
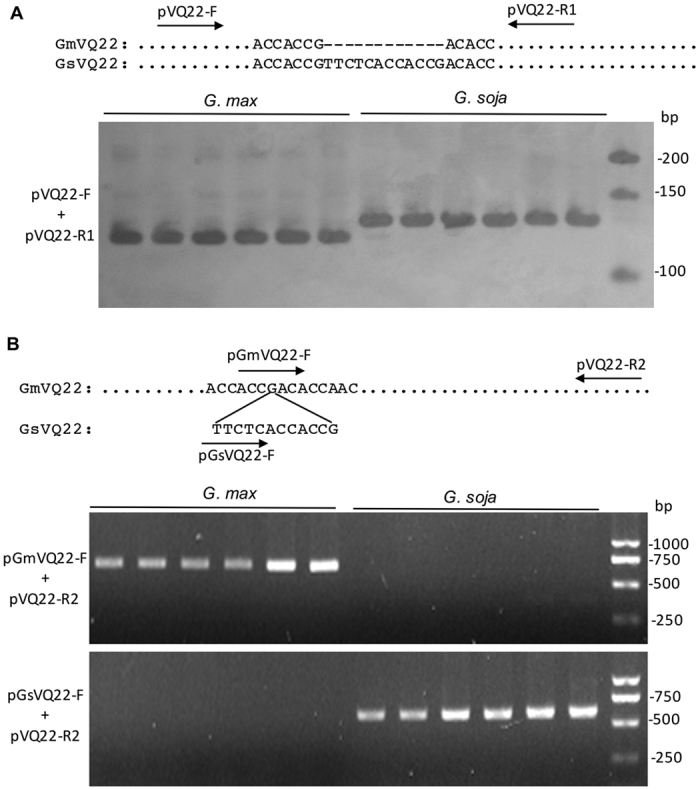
PCR-based detection of *VQ22* polymorphism in soybean. (**A**) AFLP-based detection of VQ22 and VQ22ΔV146_T149 alleles. Two primers flanking the 12-nt deletion in the VQ22ΔV146_T149 alleles (pVQ22-F and pVQ22-R1) were designed (shown in the diagram in upper panel) and used to detect AFLP in *VQ22* alleles from cultivated (*G. max*) and wild (*G. soja*) soybean. PCR products were separated on a 12% polyacrylamide gel (lower panel) (**B**). PASA-based detection of VQ22 and VQ22ΔV146_T149 alleles. Two pairs of PCR primers that specifically amplify either the *VQ22* (pGmVV22-F and pVQ22-R2) or *VQ22*Δ*V146_T149* (pGsVQ22-F and pVQ22-R2) allele (shown in the diagram in upper panel) were designed and used to detect the two alleles from cultivated (*G. max*) and wild (*G. soja*) soybean. PCR products were separated on a 0.8% agarose gel (lower panel).

Annotated genome sequence from ‘Williams 82’, a cultivated soybean line, revealed a 12 base pair (bp) deletion in *GmVQ22*, resulting in removal of 4-amino acid residues (V146, L147, T148, and T149) in the region immediately upstream of the FxxxVQxxFTG motif (VQ22ΔV146_T149) (Figs 1 and 2). Sequencing of the genes from ‘Williams 82’ confirmed the 12-bp deletion in *GmVQ22*. Interestingly, sequencing of the same gene from a wild soybean (*G. soja*) line revealed the absence of the 12-bp deletion, suggesting possible allelic polymorphism at the *VQ22* locus in the *Glycine* genus. To study the polymorphism further, we developed two PCR approaches for rapid detection of the polymorphism. We designed a pair of PCR primers for detection of the PCR-amplified fragment length polymorphism (AFLP) between GsVQ22 and GmVQ22ΔV146_T149 (Fig. 7A). We also used the method of PCR Amplification of Specific Alleles (PASA) with PCR primers that specifically amplify either the Gs*VQ22* or Gm*VQ22*Δ*V146_T149* allele (Fig. 7B)^34^. Both PCR procedures detected the polymorphism reliably and faithfully and were used to investigate their occurrence in a large collection of wild (*G. soja*), semi wild (*G. gracilis*), and cultivated (*G. max*) soybean lines. As shown in Table 1, survey of 26 wild soybean lines revealed that they all contain the GsVQ22 allele without the 12-bp deletion. By contrast, cultivated soybean lines or cultivars all contain the *GmVQ22*Δ*V146_T149* allele (Table 1). Interestingly, semi wild soybean populations contain both the *VQ22* and *VQ22*Δ*V146_T149* alleles (Table 1). Among the 20 semi wild soybean lines tested, 16 contain the *VQ22* allele, whereas the remaining 8 lines contain the *VQ22*Δ*V146_T149* allele (Table 1).

### Analysis of the upstream submotif of VQ proteins in interaction with WRKY proteins

**Figure 8:**
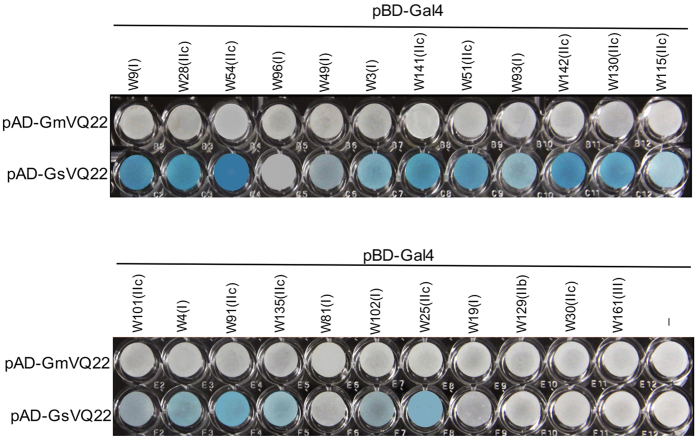
Comparison of GsVQ22 and GmVQ22 for interaction with WRKY protein in yeast cells. The Gal4 DNA BD-WRKY domain fusion bait vectors were contransformed with the activation domain (AD)-VQ fusion prey vectors into yeast cells and the transformant cells were assayed for *LacZ* reporter gene expression. The empty pBD prey vector was used as negative control. The specific subfamilies to which the tested WRKY proteins belong to are indicated in parentheses.

Even though the four amino acid residues deleted in VQ22ΔV146_T149 are located outside the FxxxVQxxhTG motif, they are immediately upstream of the highly conserved motif and, therefore, could affect binding to WRKY proteins. To test the possibility, we fused both the GsVQ22 and GmVQ22ΔV146_T149 genes with the AD of the Y2H prey vector pAD-Gal4 and cotransformed into yeast cells with the pBD-Gal4-WRKY fusion constructs from 23 soybean WRKY genes. As shown in Fig. 8, GsVQ22 interacts with 18 of the 20 tested Group I and Group IIc WRKY proteins, albeit with varying intensities based on the β-galactosidase activity. On the other hand, no interaction was detected between GsVQ22 and the three Group IIb, IIe, or III WRKY proteins (Fig. 8). Thus, like other VQ proteins, GsVQ22 interacts only with Group I and Group IIc WRKY proteins. By contrast, when the pAD-GmVQ22ΔV146_T149 fusion vector was cotransformed into yeast cells with the 23 pBD-Gal4-WRKY bait vectors, none of them displayed detectable β-galactosidase activity (Fig. 8), indicating that deletion of the four amino acid residues in GmVQ22ΔV146_T149 completely abolishes its binding to WRKY proteins.

**Figure 9:**
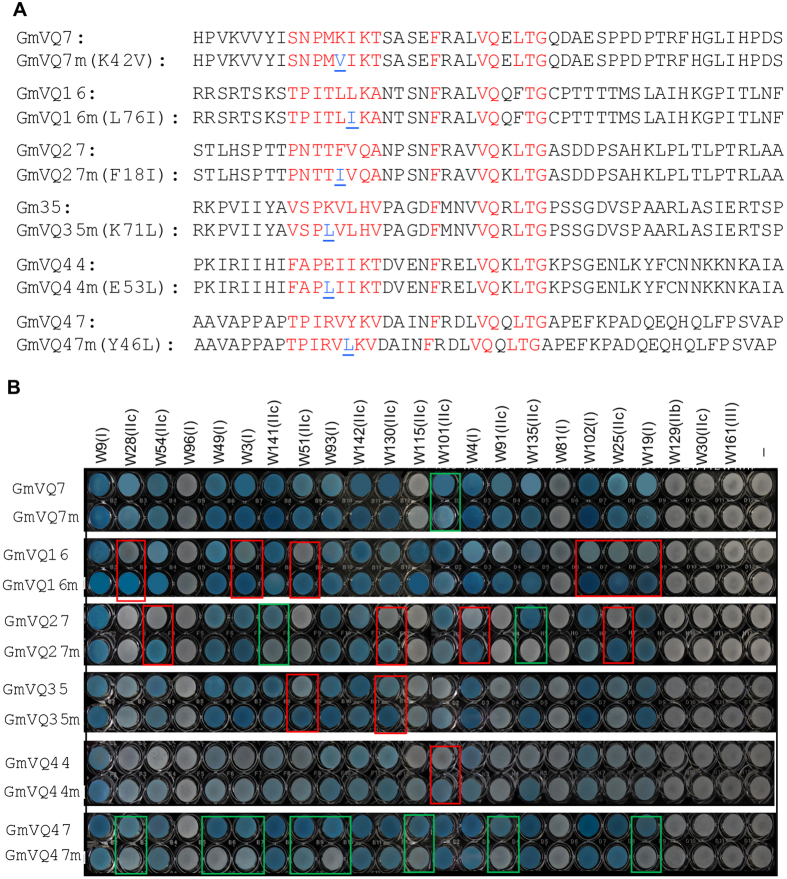
Effects of single amino acid substitutions in the upstream submotif of VQ proteins on binding to WRKY proteins. (**A**) Single amino acid substitutions in the upstream submotifs of six GmVQ proteins. Only the sequences of the extended VQ motifs are shown. The amino acid residues for the upstream submotifs and the FxxVQxxhTG core motifs are in red. The changed amino acid residues in mutated (m) VQ proteins in their upstream submotifs are in blue. (**B**) Interaction of wild-type GmVQ and mutated (m) GmVQ proteins with GmWRKY proteins in yeast cells. The Gal4 DNA BD-WRKY domain fusion bait vectors were co-transformed with the activation domain (AD)-VQ fusion prey vectors into yeast cells and the transformant cells were assayed for *LacZ* reporter gene expression. The empty pBD prey vector (−) was used as negative control. The specific subfamilies to which the tested WRKY proteins belong to are indicated in parentheses.

The complete loss of binding to WRKY proteins by GmVQ22ΔV146_T149 suggests a critical role of the upstream submotif of VQ proteins in their recognition of the WRKY domains of Group I and IIc WRKY proteins. However, the loss of WRKY-binding activity in GmVQ22ΔV146_T149 could also be due to the effects of the deletion of four amino acid residues on the folding and conformation of the protein. Indeed, phylogenetic analysis and sequence alignment have revealed that the amino acid residues in the upstream submotif are not highly conserved but largely clade-specific (Fig. 2). These observations suggest that the upstream sequence is not the core motif essential for WRKY-binding but could be required for clade-specific properties of VQ proteins. To distinguish between the two possibilities, we selected six GmVQ proteins (GmVQ7, GmVQ16, GmVQ27, GmVQ35, GmVQ44 and GmVQ47) from different clades in the phylogenetic tree with strong binding to Group I and IIc WRKY proteins and changed a single amino acid residue in the upstream clade-specific submotif using overlapping PCR (Fig. 9A). The mutated genes were fused with the AD of the pAD-Gal4 prey vector and cotransformed into the yeast cells with pBD-Gal4-WRKY vectors for protein interaction assays. As shown in Fig. 9B, change of a single amino acid residue in the upstream submotif of the VQ proteins did not abolish their binding to WRKY proteins. However, their binding specificity to a number of tested WRKY proteins was altered by these single amino acid substitutions. For example, the L76I substitution of GmVQ16 increased its binding to a number of Group I and IIc WRKY proteins (Fig. 9). By contrast, the Y46L substitution in the upstream motif of GmVQ47 resulted reduced binding to a number Group I and IIc WRKY proteins (Fig. 9). Interestingly, the F18I substitution in the upstream motif of GmVq18 increased binding to four Group I and IIc WRKY proteins but reduced binding to two other WRKY proteins. Single amino acid substitutions in the other three GmVQ proteins also alters their binding to WRKY proteins, although the number of affected WRKY proteins was smaller (Fig. 9).

### Functional analysis of soybean VQ22 variants

**Figure 10:**
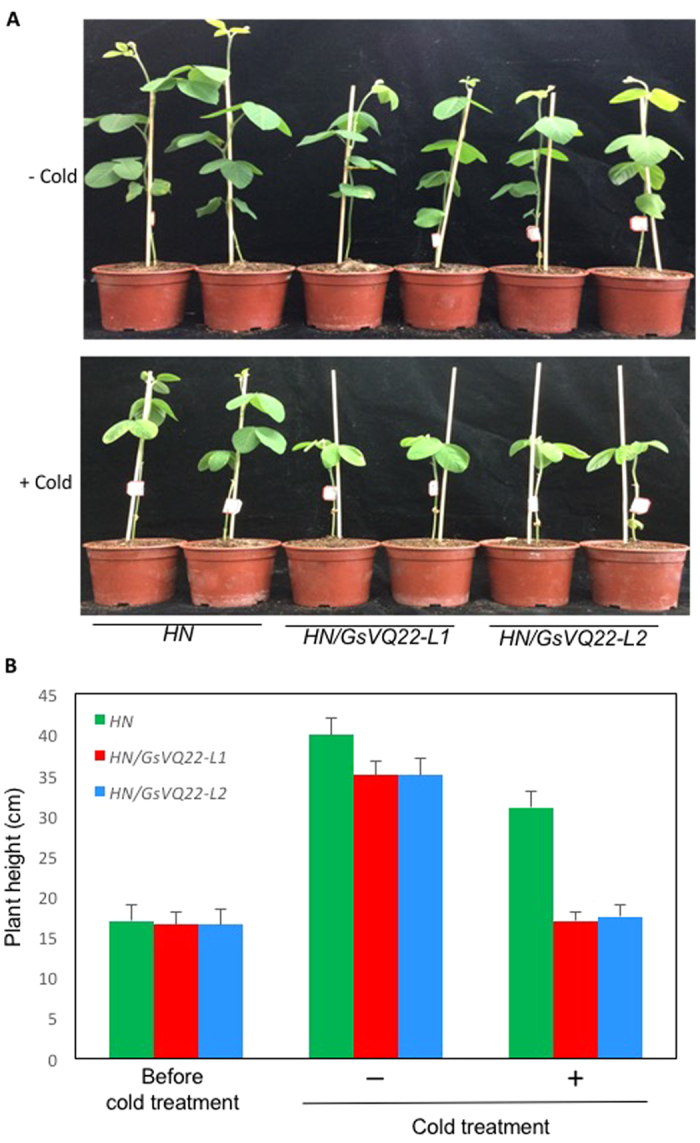
Growth of transgenic GsVQ22 soybean plants. (**A**) Growth of transgenic GsVQ22 soybean plants with or without cold treatment. Untransformed (HN) and two independent transgenic GsVQ22 (HN/GsVQ22) soybean plants were grown in a growth room at 24 °C with a 12/12 h hour light/dark photoperiod. Two weeks old seedlings were placed in a growth chamber at 3 °C for 48 h with a 12/12 h hour light/dark photoperiod. After the cold treatment, the plants were placed in a growth chmaber at 24 °C for recovery. Pictures were taken two weeks after cold treatment. Plants without cold treatment were also show as control (upper panel). (**B**) Heights of transgenic GsVQ22 soybean plants with or without cold treatment. Growth and cold treatment were as in A. Plant heights with (+) or without (−) cold treatment (at 3 °C for 48 h) were determined after two weeks at 24 °C following the cold treatment. Heights of plants before the cold treatment are also shown.

**Figure 11:**
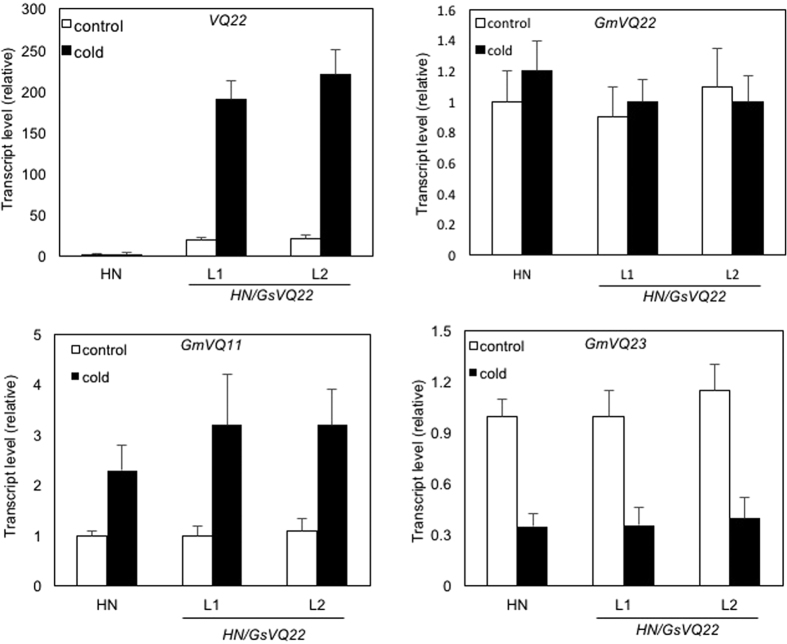
Expression of *GsVQ22*, *GmVQ22*, *GmVQ11* and *GmVQ23* in the transgenic *GsVQ22* soybean plants. Two weeks old nontransgenic (HN) and two independent lines (L1 and L2) of transgenic *GsVQ22* plants (HN/GsVQ22) were subjected to cold treatment (at 3 °C for 48 hours). Leaf samples were collected before and after cold treatment for RNA isolation and qRT-PCR analysis of transcript levels for *VQ22* (*GsVQ22*+*GmVQ22*), *GmVQ11* and *GmVQ23* using gene-specific primers.

Deletion of four amino acid residues in the upstream submotif and abolishment of WRKY-binding activity of GmVQ22 suggest that this is likely a loss-of-function mutation that might be advantageous and positively selected for during domestication. To examine this, we tried to express the wild soybean GsVQ22 in cultivated soybean to determine the effects on the transgenic soybean plants. For this purpose, we placed the *GsVQ22* coding region under the control of the CaMV *35S* promoter in the binary plant transformation vector pFGC5941. Using cotyledonary node method, we transformed the *GsVQ22* expression construct into the soybean cultivar Heinong (HN) 37 and generated eight independent transgenic lines. However, six of the eight transgenic plants grew very poorly and didn’t set seeds. The two remaining lines grew and set seeds largely normally and homozygous F3 progeny from the two independent lines were obtained. At early stages, homozygous F3 progeny from the transgenic *GsVQ22* plants were largely normal when compared with nontransgenic control plants (Supplemental Fig. 1). However, at older stages, the transgenic *GsVQ22* plants displayed a slight but significant reduction in growth. At 4-week age, the average heights of the transgenic plants were about 10–15% less than those of untransformed control plants (Fig. 10). qRT-PCR analysis showed that the *VQ22* transcript levels in the two independent transgenic lines were elevated by 10–20-fold when compared to those in untransformed HN37 plants (Fig. 11). To determine possible roles in stress tolerance, we also examined their responses to cold, heat and drought stresses but failed to observe significantly altered phenotypes. Interestingly, we consistently observed that reduced growth of the transgenic plants was enhanced after cold treatment. In these experiments, we first subjected 12-days old transgenic and control plants to 48-hour cold treatment at 3 °C. After the cold treatment, all the plants were moved back to a growth room at 24 °C for recovery of growth. This cold treatment significantly reduced the growth of nontransgenic control plants as indicated from the ~20% reduction in plant heights after 2-week recovery at 24 °C when compared to those of unstressed plants (Fig. 10). Interestingly, the cold treatment reduced the growth of transgenic *GsVQ22* plants to a larger extent as these plants displayed almost no increase in their height after 2 week recovery at 24 °C after the cold treatment (Fig. 10). As a result, the heights of the cold-treated transgenic *GsVQ22* plants were only about 60% of cold-treated nontransgenic plants after recovery for 2 weeks at 24 °C (Fig. 10). Thus, *GsVQ22* had an inhibitory effect on soybean growth and this inhibitory effect was strengthened by cold treatment.

To understand how the inhibitory effect of GsVQ22 on soybean growth was temperature-sensitive, we analyzed the effects of cold treatment on expression of *VQ22* in both transgenic and non-transgenic control plants. As described earlier, prior to cold treatment, transgenic *GsVQ22* plants contained the levels of *VQ22* transcripts 10–20 times higher than those in nontransgenic plants (Fig. 11). Interestingly, after 48-cold cold treatment, transgenic *GsVQ22* plants had an ~10-fold increase in *VQ22* transcripts, while nontransgenic plants displayed no such increase (Fig. 1). As a result, the *VQ22* transcript levels in the transgenic *GsVQ22* plants were ~200 times higher than those in nontransgenic plants after the cold treatment (Fig. 11). Thus, there was a correlation between the degrees of growth inhibition and the *VQ22* transcript levels. Without cold treatment, the transcript levels of *VQ22* were elevated by 10–20-fold in the transgenic *GsVQ22* plants (Fig. 11), leading to slight but significant inhibition of growth (Fig. 10). With cold treatment, the transcript levels of *VQ22* in the transgenic *GsVQ22* plants were further elevated by ~10-fold (Fig. 11), resulting in even more inhibition of the growth of the transgenic plants (Fig. 10).

In the transgenic *GsVQ22* plants, the *GsVQ22* transgene is almost identical to the endogenous *GmVQ22* gene in DNA sequence except for the 12-bp deletion. Both the *GsVQ22* and *GmVQ22* genes are also highly homologous to *GmVQ11* and *GmVQ23* and, as a result, there could be cross-amplification during qRT-PCR analysis of the *VQ22* transcripts. To determine whether the large induction of *VQ22* transcripts upon cold treatment is due to increased expression of the endogenous *GmVQ22*, *GmVQ11* and *GmVQ23* genes or the *GsVQ22* transgene, we designed gene-specific primers for the four VQ genes and used them for qRT-PCR analysis of gene-specific transcripts. qRT-PCR analysis using gene-specific primers revealed no significant difference between nontransgenic plants and transgenic *GsVQ22* plants for the transcript levels of the endogenous *GmVQ11*, *GmVQ22* and *GmVQ23* genes (Fig. 11). Furthermore, cold treatment did not substantially affect the transcript levels of the three endogenous VQ genes (Fig. 11). By contrast, when using *GsVQ22* transgene-specific primers, we observed >10-fold increase in its transcripts in the transgenic *GsVQ22* plants after cold treatment (Supplemental Fig. 2). Thus, the expression levels of the *GsVQ22* transgene in the two transgenic *GsVQ* lines were modest under normal condition but were substantially induced by cold temperature. The cold-induced elevation in expression of the *GsVQ22* transgene was associated with increased growth inhibition of the transgenic *GsVQ22* plants.

## Discussion

### Structures and functions of soybean VQ protein family

Soybean VQ gene family contains 74 members, which is substantially larger than those from several other plants including Arabidopsis^18^, rice^20^, Chinese cabbage^29^ and grapevine^30^, where genome-wide identification of their VQ gene families have been reported. The large VQ gene family in soybean is likely related to the polyploid nature of soybean, which has undergone at least two rounds of large-scale duplication at approximately 14 and 42 million years ago^33^. Indeed, a majority of soybean VQ genes have one or more close homologs on different chromosomes (Fig. 2), suggesting that they resulted mostly from duplication of large chromosome regions or whole chromosomes. Despite the expansion of the gene family, all identified soybean VQ proteins contain the FxxxVQxxhTG motif flanked by divergent sequences as found with VQ proteins from other plant species (Figs 1 and 2). Y2H assays with 20 soybean VQ proteins from different subfamilies (or clades on the phylogenetic tree) showed that they all bind only to the C-terminal WRKY domains of Group I WRKY proteins and the single WRKY domain of Group IIc WRKY proteins (Fig. 3). Previously we have shown that the conserved V and Q residues in the FxxxVQxxhTG motif of Arabidopsis SIB1/VQ23 are required for its interaction with WRKY33, suggesting that the FxxxVQxxhTG motif is the core motif for WRKY binding^10^. The fact that all identified VQ proteins from distantly related plant species contain the highly conserved FxxxVQxxhTG motif, despite the great expansion of the gene family and the highly divergent flanking sequences, strongly suggests that binding of WRKY proteins remains the most important or even perhaps the central activity of plant VQ proteins.

Plant WRKY proteins have a predominant role in plant responses to biotic and abiotic stresses^3^,^5^,^35^. As their interacting proteins, plant VQ proteins are likely to be important regulators of plant disease resistance and stress tolerance as well. Molecular genetic analysis of a number of Arabidopsis VQ genes support the critical roles of VQ proteins in plant stress responses^10^,^18^,^19^,^24^,^26^,^28^,^36^. In this study, we have provided further evidence for roles of VQ proteins in defense responses of soybean plants. First, expression of a substantial percentage of soybean VQ genes was responsive to a number of phytohormones associated with plant defense and stress responses (Fig. 4). In particularly, a large number of soybean VQ genes were responsive to SA, an important defense signal in plants (Fig. 4). Previously, a large percentage of Arabidopsis WRKY and VQ genes are responsive to SA as well^4^,^18^. Survey of Arabidopsis WRKY and VQ gene promoters have further revealed strong enrichment of TTGAC W-box elements, suggesting extensive auto- and cross-regulation of Arabidopsis WRKY and VQ genes^4^,^18^. Survey of soybean VQ gene promoters likewise revealed strong enrichment of W boxes as well (data not shown). These findings indicate that auto- and cross-regulation is a conversed regulatory mechanism of plant WRKY and VQ genes, leading to formation of both positive or negative feedback loops and, consequently, rapid induction or repression of the genes under stress conditions. Second, overexpression of both soybean *GmVQ35* and *GmVQ47* caused enhanced susceptibility of transgenic Arabidopsis plants to *Botrytis* (Fig. 6A). Overexpression of *GmVQ47* also reduced heat tolerance of transgenic plants (Fig. 6B). Similarly, Arabidopsis contains not only positive but also negative VQ regulators of plant stress responses^10^,^18^,^19^,^24^,^26^,^28^,^33^. The opposite roles of VQ proteins in plant stress responses could be at least in part mediated by their opposite effects on interacting WRKY proteins. Thus, while Arabidopsis SIB1/AtVQ23 proteins enhance the DNA-binding activity of the interacting AtWRKY33 protein, other VQ proteins may inhibit the DNA-binding activity of their interacting WRKY proteins. Furthermore, some Arabidopsis VQ proteins act as transcriptional activators while others function as transcriptional repressors in plant cells^19^,^28^. Therefore, a VQ protein could positively or negatively regulate the expression of the target genes of their interacting WRKY proteins by altering their DNA-binding and transcription-regulating activities. Coordinated action of both positive and negative VQ transcription regulators could potentially provide a mechanism for tight regulation and fine-tuning of genes associated with plant stress responses.

Although WRKY proteins have been established to play a role in specific developmental processes such as trichome and seed development, a broad role of plant WRKY proteins in regulation of plant flowering has yet to emerge. Interestingly, a number of plant VQ proteins have been show to alter flowering time when overexpressed in Arabidopsis. Overexpression of Arabidopsis AtVQ29 substantially delayed Arabidopsis flowering^18^. By contrast, overexpression of soybean GmVQ43 and GmVQ62 promoted flowering when overexpressed in Arabidopsis (Fig. 5B). Interestingly, a number of soybean WRKY proteins were also able to alter flowering time in Arabidopsis plants^37^,^38^. Further analysis revealed that a number of Arabidopsis flowering time genes including a substantial number of W boxes in their elements that are recognized by the expressed soybean WRKY proteins^38^. Even though these altered flowering phenotypes were resulted from overexpression of transgenes, they nevertheless provide clues to perhaps an important role of plant WRKY and VQ proteins in the regulation of plant development, particularly under stress conditions.

### Structural elements of VQ motifs affecting WRKY-binding specificity

Arabidopsis contains 34 VQ genes and 32 Group I and IIc WRKY genes^18^. In soybean, there are 74 VQ genes and 72 Group I and IIc WRKY genes^39^. Despite the similar numbers of VQ and Group I and IIc WRKY genes, the interaction patterns clearly indicated that they are not one-to-one partners. A single VQ protein often interacts with multiple WRKY proteins and vice versa (Fig. 3)^18^. Obviously, the specific biological functions of a VQ protein requires not only the conserved VQ motif for binding of WRKY proteins but also the flanking sequences, which are highly divergent among VQ proteins. The divergent flanking sequences of a VQ protein could determine or influence its subcellular localization, protein stability and interacting partners, thereby affecting how a specific WRKY/VQ complex regulates transcription of target genes and associated biological processes. In addition, the promoter sequence of a VQ gene would determine its spatial and temporal expression patterns and consequently determine which co-expressed WRKY proteins the VQ protein will be able to partner with.

Despite the facts that all identified VQ proteins contain the FxxxVQxxhTG motif and they all appear to interact only with Group I and IIc WRKY proteins, there is difference in WRKY-binding specificity among Arabidopsis and soybean VQ proteins (Fig. 3)^18^. While some VQ proteins are able to interact with a large number of Group I and IIc proteins, other VQ proteins display very high binding specificity. To understand the structural basis of the WRKY-binding specificity of VQ proteins, we have analyzed a region immediately upstream of the FxxxVQxxhTG motif in several soybean VQ proteins and provided new evidence for a role of the upstream submotif in determining WRKY-VQ binding specificity. First, we have previously shown that mutating the V and Q residues in the FxxxVQxxhTG motif completely abolish its binding to a WRKY protein, indicating that this short VQ motif is the core element in binding of WRKY proteins^10^. On the other hand, change of a single amino acid residue in the upstream submotif does not completely abolish WRKY protein binding (Fig. 9). However, deletion of four amino acid residues in the same region in GmVQ22 resulted in a complete loss of binding to WRKY proteins (Fig. 8). These observations suggest that unlike the FxxxVQxxhTG core motif essential for binding WRKY proteins, the upstream submotif is likely to be a modulating element of the core binding motif, affecting WRKY-VQ protein binding affinity and specificity. Consistent with this interpretation, we were able to alter their binding specificity to Group I and IIc WRKY proteins by changing a single amino acid residue in the upstream submotif of several soybean VQ proteins (Fig. 9). Alteration in binding specificity to group I and IIc WRKY proteins as a result of a single amino acid change was observed for all six soybean VQ proteins but were particularly strong and extensive with GmVQ27 and GmVQ47, in which an aromatic amino acid residue (F or Y) was changed to a non-aromatic residue (I or L) (Fig. 9). This would argue for a role of steric hindrance in determining the VQ-WRKY binding specificity. Alteration in binding specificity was also very strong with GmVQ16 when a leucine residue was changed to an isoleucine residue (GmVQ16L76I) (Fig. 9), which is surprising given the similarity between the two amino acid residues. To provide clues to the questions, we have analyzed the secondary structures of the VQ domains using programs for protein structure prediction. The FxxxVQxxhTG motif is predicted to be largely an α-helical structure, whereas the upstream submotif is predicted to display largely a β-sheet structure. The β-sheet secondary structure of the upstream submotifs of different VQ proteins also appear to be highly conserved despite their variation in amino acid residues. Interestingly, even though leucine and isoleucine both have branched and strongly hydrophobic four-carbon side chains, leucine is a strong α-helix forming residue while isoleucine strongly favors the β-conformation^40^,^41^,^42^, which the upstream submotif of VQ protein probably adopts and may explain the overall enhancement of binding of GmVQ16L76I to a number of Group I and IIc WRKY proteins over wild-type GmVQ76.

Identification of the upstream submotif of the VQ proteins as a critical factor for the VQ-WRKY binding specificity could be very useful, particularly if the crystal structure of a VQ/WRKY protein complex becomes available. Like the VQ motif of VQ proteins, the C-terminal WRKY domain of Group I WRKY proteins and the single WRKY domain of Group IIc WRKY proteins consist of invariable WRKYGQK and zinc finger residues as well as variable residues, some of which are likely in direct contacts with the VQ motifs and determine their binding specificity to VQ proteins. Once the specific motifs from both WRKY and VQ proteins that determine their binding specificity are identified, it should be possible to elucidate the underlying structural basis with a combined approach of structural biology, site-directed mutagenesis and bioinformatics. This information could then be used to study how plant VQ proteins and their interacting WRKY proteins proliferate and evolve to form new interacting complexes with distinct biological functions. This knowledge could also be used to develop novel VQ proteins that target specific WRKY proteins for manipulation of their molecular properties and ultimately the associated biological processes.

### Roles and evolution of soybean VQ22 variants

Molecular genetic analysis of crop domestication has been a topic of active research not only for addressing the basic questions on the geographical origin and the events of domestication for a given crop species but also for understanding the specific molecular changes underlying important crop traits during domestication and subsequent selection, which can be highly useful for further crop improvement. In our study of soybean VQ gene family, we have discovered that soybean *VQ22* gene has apparently undergone a genetic change during soybean evolution. While wild soybean (*G. soja*) lines contain a VQ22 protein with a functional VQ motif capable of binding WRKY proteins, cultivated soybean (*G. max*) lines and cultivars have a VQ22 protein (VQ22ΔV146_T149) incapable of binding WRKY proteins due to deletion of four amino acid residues in the region immediately upstream of the FxxxVQxxhTG motif (Table 1). In different semi-wild soybean lines (*G. gracilis*), interestingly, both forms of VQ22 are found, with a majority of them containing the GsVQ22 form as in wild soybean lines (Table 1). Semi-wild soybean is an important intermediary type of soybean with both wild and domesticated soybean characteristics. Recent genetic and genomic analyses have revealed that semi-wild soybean is probably not an intermediate transition type of soybean domestication^43^,^44^. Instead, semi-wild soybean was likely resulted from the hybridization of wild and domesticated soybean^43^,^44^. Accordingly, deletion of the 4 amino acid residues in VQ22 likely occurred during soybean domestication or subsequent selection and both the *VQ22* and *VQ22*Δ*V146_T149* alleles occur in semi-wild soybean due to the hybridization between the *GsVQ22*-containing wild soybean and *GmVQ22*Δ*V146_T149*-containing cultivated soybean.

Deletion of the four amino acid residues in the upstream submotif of VQ22 abolishes its activity to bind WRKY protein (Fig. 8) and, therefore, is likely to be a loss-of-function mutation, although a possible new activity of VQ22ΔV146_T149 cannot be completed excluded. For functional analysis of the deletion mutation of *VQ22*, we have transformed the *GsVQ22* gene into a cultivated soybean cultivar and observed significant reduction in growth, particularly with increased expression after cold treatment, in the transgenic plants (Figs 10 and 11). *GsVQ22*-mediated inhibition of growth would be undesirable for maximizing plant biomass and seed yield of soybean and a loss-of-function mutation of *GsVQ22* would be advantageous and therefore could have been positively selected and fixed in domesticated soybean. The absence of the loss-of-function mutation of *GsVQ22* in wild soybean, despite its negative effect on plant growth, could be due to the association of the gene with other beneficial traits such as disease resistance and stress tolerance that are particularly important to wild soybean.

One intriguing discovery from the study is that expression of the *GsVQ22* transgene under control of the constitutive *CaMV 35S* promoter was elevated ~10-fold after cold treatment (Fig. 11), which led to further inhibition of growth of the transgenic plants (Fig. 10). Two independent transgenic lines displayed similar cold-responsiveness of the transgene induction by cold temperature, suggesting that it is probably not due to specific DNA elements at the insertion sites of the transgene construct in the transgenic plants. More likely, cold-induced expression of the *GsVQ22* transgene was a result of reduced silencing of the transgene expression at low temperatures. Due to its inhibitory effect on growth, overexpression of *GsVQ22* at high levels would have detrimental effects on transgenic plants and this might explain why 6 of the 8 transgenic lines generated from the study had poor growth and no seed setting. In the two surviving lines with modest levels of growth inhibition, the *GsVQ22* transcript levels were elevated by 10–20-fold (Fig. 11), which is not particularly high for the strong *CaMV 35S* promoter. The relatively low levels of the *GsVQ22* transgene expression in the two transgenic lines could be due to partial silencing of the transgene, which is common for transgenes driven by a strong promoter^45^. Upon cold treatment, silencing of the *GsVQ22* transgene was reduced, leading to increased expression of the transgene and enhanced growth inhibition of transgenic plants (Figs 10 and 11). Temperature-sensitive gene silencing has been reported in both animals and plants^46^,^47^,^48^,^49^,^50^,^51^. In addition, the general levels of DNA methylation are reduced in plants at low temperature and gene silencing is less efficient^52^. In addition, the Antirrhinum T*am3* transposon is excised at 15 °C, but not at 25 °C, and the transposition correlates with reduced methylation status of mobile element^53^,^54^,^55^. Further analysis will be necessary to determine whether increased expression of the transgenic *GsVQ22* gene in transgenic plants is resulted from reduced silencing at the transcriptional or post-transcriptional levels.

## Methods

### Plant materials and growth conditions

Soybean (*Glycine max* cv ‘Williams 82’ and ‘Heinong 37’) and Arabidopsis were grown in a greenhouse or growth room at 24 °C with a photoperiod of 12 h.

### Identification and phylogenetic analysis of soybean VQ proteins

Published Arabidopsis VQ protein sequences were used in BLASTp searches for GmVQ proteins in the soybean genome (http://www.phytozome.net, Glycine max Wn82.a2.v1). All final data sets were downloaded in May. 2016. The Pfam database was employed to ascertain if the candidate proteins contained features typical of VQ proteins. The phylogenetic tree based on complete amino acid sequences of VQ proteins from soybean was inferred using the neighbor-joining method. Phylogenetic analyses were conducted in MEGA6. Bootstrap values from 1,000 replicates were used to assess the robustness of the tree.

### qRT-PCR analysis of VQ gene expression

Soybean tissue samples were lyophilized and stored at −80 °C until use. Total RNA was isolated from soybean tissues using the Trizol reagent according to the supplier’s instruction. Extracted RNA was treated with DNase to remove contaminating DNA and reverse transcribed using the ReverTran Ace^®^ qPCR RT kit (Toyobo) for reverse transcriptase-PCR. qRT-PCR was performed with an StepOnePlus™ Real-Time PCR System (ABI). PCRs were performed using the SYBR^®^ Green qPCR Master Mixes (Takara) and gene-specific primers (Supplemental Table 2). The PCR conditions consisted of denaturation at 95 °C for 1 min, followed by 40 cycles of denaturation at 95 °C for 5s, 55 °C annealing for 30s and extension at 72 °C for 30s. Melt curve analysis was performed on the end products of PCR, to determine the specificity of reactions. Relative quantification of gene expression was calculated according to the ΔΔCt method. The soybean actin gene (Gm18g52780) was used as internal control. The heatmap was visualized using Heatmapper Plus tool at the Bio-Array Resource for Plant Functional Genomics.

### Analysis of protein-protein interactisn using yeast two-hybrid assays

Interactions between WRKY and VQ proteins were assayed using Gal4-based two-hybrid system in yeast (*Saccharomyces cerevisiae*). pAD-Gal-VQ and pBD-Gal-WRKY fusion constructs were generated from PCR-amplified coding sequences for GmVQ genes using the gene-specific primers (Supplemental Tables 3 and 4). Mutant VQ genes were generated with overlapping PCR using gene-specific primers (Supplemental Fig. 6). The prey and bait plasmids were transformed to yeast strain YRG-2. After two days culture, the transformants were picked from the selection plates and inoculated into snap-cap tubes containing 3 ml of selective medium. Cultures were collected when the absorbance A_546_ reached 0.8–1. Centrifugation at 2000 × g for 5 min and the supernatant was discarded. Cell lysis was performed by two freeze-thaw cycles (3 min in liquid nitrogen, 3 min in a 37 °C water bath). Pellets were subsequently resuspended in 20 μl water, transferred to a transparent flat bottom 96-well microplate, mixed with 100 μL phosphate-buffered saline buffer, pH 7.4, containing 500 μg/mL X-gal, 0.5% (w/v) agarose, and 0.05% (v/v) β-mercaptoethanol and incubated at 28 °C^56^.

### Generating transgenic GmVQ-expressing Arabidopsis plants

For generating transgenic VQ overexpression lines, the full-length coding sequences for VQ genes were amplified using gene-specific primerers (Supplemental Table 5). The amplified fragments were digested using appropriate restriction enzymes and inserted into the plant transformation vector pFGC5941 containing the CaMV35S promoter. The fused plasmids were transformed into Col-0 wild-type plants using the Agrobacterium-mediated floral-dip procedure^57^. Transformants were identified for resistance to basta herbicide. Transgenic plants overexpressing the transformed GmVQ transgenes were identified using qRT-PCR.

### Analysis of disease resistance and heat tolerance of Arabidopsis plants

For analyzing resistance of trangenic Arabidopsis plants to *Botrytis cinerea*, fully expanded rosette leaves from 5–6-week-old soil-grown plants were inoculated by adding 5 μl of a *Botrytis* spore suspension at a density of 5 × 10^5^ spores ml^−1^ and kept at high humidity in Petri dishes. Photographs of representative leave were taken 4 days after inoculation^58^. For analying heat tolerance of transgenic arabidopsis plants, 5–6-week-old soil-grown plants were placed in growth chamber 45 °C for 10 hours. The plants were then moved back to a growth room at 25 °C and pictures of representative plants were taken after 4-day recovery.

### PCR detection of the VQ22ΔV146_T149 deletion in soybean VQ22 gene

Soybean genomic DNA was extracted and used for PCR delection of the VQ22ΔV146_T149 deletion in soybean VQ22 gene in a wide range of wide, semi-wild and cultivated soybean lines using two procedures: PASA^34^ and AFLP. Detection with the PASA method used primers specific for GsVQ22 (5′-TGCACCCACCACCGTTCT-3′ and 5′-AGGGGAATTTCCATTTGCAT-3′) and GmVQ22 (5′-GGCGTGCACACACCACAGA-3′ and 5′-AGGGGAATTTCCATTTGCAT-3′). Detection of AFLP between *GsVQ22* and *GmVQ22* was performed using the primers 5′-CACGACAACATTAATGATAATTCAAT-3′ and 5′-AGTTGTTGGTGTCGGTGGT-3′.

### Soybean transformation of the *GsVQ22* gene

The intronless *GsVQ22* gene was PCR-amplified from the genomic DNA isolated from a wild soybean line with gene-specific primers (5-AGCCTCGAGATGGACTCTGGTAACAGTGGA-3 and 5-AGCTCTAGATCACACCCCACGCGAGATCATATT-3). The amplified fragment cloned into the plant transformation vector pFGC5941 containing the *CaMV 35S* promoter. The *GsVQ22* gene was transformed into the soybean cultivar Heinong 37 using Agrobacterium-mediated cotyledonary-node transformation method^59^,^60^. Transgenic soybean plants were verified by resistance to the Basta herbicide and PCR analysis of both the *Bar* and *GsVQ22* genes using gene-specific primers (*Bar*: 5′- GTACCGGCAGGCTGAAGTC -3 and 5-GCACCATCGTCAACCACTAC-3′; *GsVQ22*: 5′-TTTCATTTGGAGAGGACACG-3 and 5-GGTGAGAGATGGAACCGAAA-3′). Homozygous T_3_ transgenic plants were used for qRT-PCR analysis of *VQ* expression and for analysis of growth phenotypes.

## Additional Information

**How to cite this article**: Zhou, Y. *et al*. Structural and Functional Characterization of the VQ Protein Family and VQ Protein Variants from Soybean. *Sci. Rep*. **6**, 34663; doi: 10.1038/srep34663 (2016).

## Supplementary Material

Supplementary Information

## Figures and Tables

**Table 1:** Genotyping of the *VQ22* alleles in a wide range of soybean lines.

Line name	species	*VQ22* allele^a^	Line name	species	*VQ22* allele^a^
PI81762	*G. soja*	*VQ22*	PI81763	*G. gracilis*	*VQ22*
PI101404B	*G. soja*	*VQ22*	PI81771	*G. gracilis*	*VQ22*
PI342618B	*G. soja*	*VQ22*	PI81772	*G. gracilis*	*VQ22*
PI342620A	*G. soja*	*VQ22*	PI86046	*G. gracilis*	*VQ22*
PI342622A	*G. soja*	*VQ22*	PI135590	*G. gracilis*	*VQ22*
PI406684	*G. soja*	*VQ22*	PI232987	*G. gracilis*	*VQ22*
PI407288	*G. soja*	*VQ22*	PI232989	*G. gracilis*	*VQ22*
PI407289	*G. soja*	*VQ22*	PI232992	*G. gracilis*	*VQ22*
PI407296	*G. soja*	*VQ22*	PI417138	*G. gracilis*	*VQ22*
PI407297	*G. soja*	*VQ22*	PI417139	*G. gracilis*	*VQ22*
PI407298	*G. soja*	*VQ22*	PI291309C	*G. gracilis*	*VQ22*
PI407299	*G. soja*	*VQ22*	PI326580	*G. gracilis*	*VQ22*
PI424004A	*G. soja*	*VQ22*	PI253651C	*G. gracilis*	*VQ22*Δ*V146_T149*
PI424004B	*G. soja*	*VQ22*	PI291275	*G. gracilis*	*VQ22*Δ*V146_T149*
PI440913A	*G. soja*	*VQ22*	PI416762	*G. gracilis*	*VQ22*Δ*V146_T149*
PI464890A	*G. soja*	*VQ22*	PI88788	*G. gracilis*	*VQ22*Δ*V146_T149*
PI464891B	*G. soja*	*VQ22*	Peking	*G. gracilis*	*VQ22*Δ*V146_T149*
PI464891C	*G. soja*	*VQ22*	PI209332	*G. gracilis*	*VQ22*Δ*V146_T149*
PI468916	*G. soja*	*VQ22*	PI90763	*G. gracilis*	*VQ22*Δ*V146_T149*
PI479744	*G. soja*	*VQ22*	PI89772	*G. gracilis*	*VQ22*Δ*V146_T149*
PI479746B	*G. soja*	*VQ22*	Williams 82	*G. max*	*VQ22*Δ*V146_T149*
PI479748	*G. soja*	*VQ22*	Heinong 37	*G. max*	*VQ22*Δ*V146_T149*
PI479750	*G. soja*	*VQ22*	Jack	*G. max*	*VQ22*Δ*V146_T149*
PI483460B	*G. soja*	*VQ22*	F002-4-4	*G. max*	*VQ22*Δ*V146_T149*
PI507581	*G. soja*	*VQ22*	Zhongdou27	*G. max*	*VQ22*Δ*V146_T149*
PI522182B	*G. soja*	*VQ22*	ZDD5957	*G. max*	*VQ22*Δ*V146_T149*
PI65388	*G. gracilis*	*VQ22*	A8901	*G. max*	*VQ22*Δ*V146_T149*
PI79593	*G. gracilis*	*VQ22*	Wupiqingren	*G. max*	*VQ22*Δ*V146_T149*
PI79648	*G. gracilis*	*VQ22*	L21778	*G. max*	*VQ22*Δ*V146_T149*
PI79727	*G. gracilis*	*VQ22*	L6326	*G. max*	*VQ22*Δ*V146_T149*

^a^The alleles of the VQ22 locus were determined by both AFLP- and PASA-based procedures. The *VQ22* allele encodes a normal VQ22 protein with an intact VQ motif, whereas the *VQ22*Δ*V146_T149* allele encodes a VQ22 protein with the deletion of four amino acid residues in the upstream submotif of the VQ22 protein.
