# Supplementary material for: Structural and Functional Characterization of the VQ Protein Family and VQ Protein Variants from Soybean
Source: Sci Rep. 2016 Oct 6;6:34663. doi: 10.1038/srep34663 (PMC5052590; doi:10.1038/srep34663)
Supplement: Supplementary Information [file srep34663-s1.pdf]

SUPPLEMENTARY INFORMATION

Structural and Functional Characterization of the VQ Protein Family and VQ Protein Variants from Soybean

Yuan Zhou[1], Yan Yang[1], Xinjian Zhou[1], Yingjun Chi[1], Baofang Fan[2] and Zhixiang Chen[1, 2,*]

[1]Department of Horticulture, Zijingang Campus, 866 Yuhangtang Road, Zhejiang University, Hangzhou, 310058, China
[2]Department of Botany and Plant Pathology, 915 W. State Street, Purdue University, West Lafayette, IN 47907, USA

*Corresponding author (Email: zhixiang@purdue.edu)

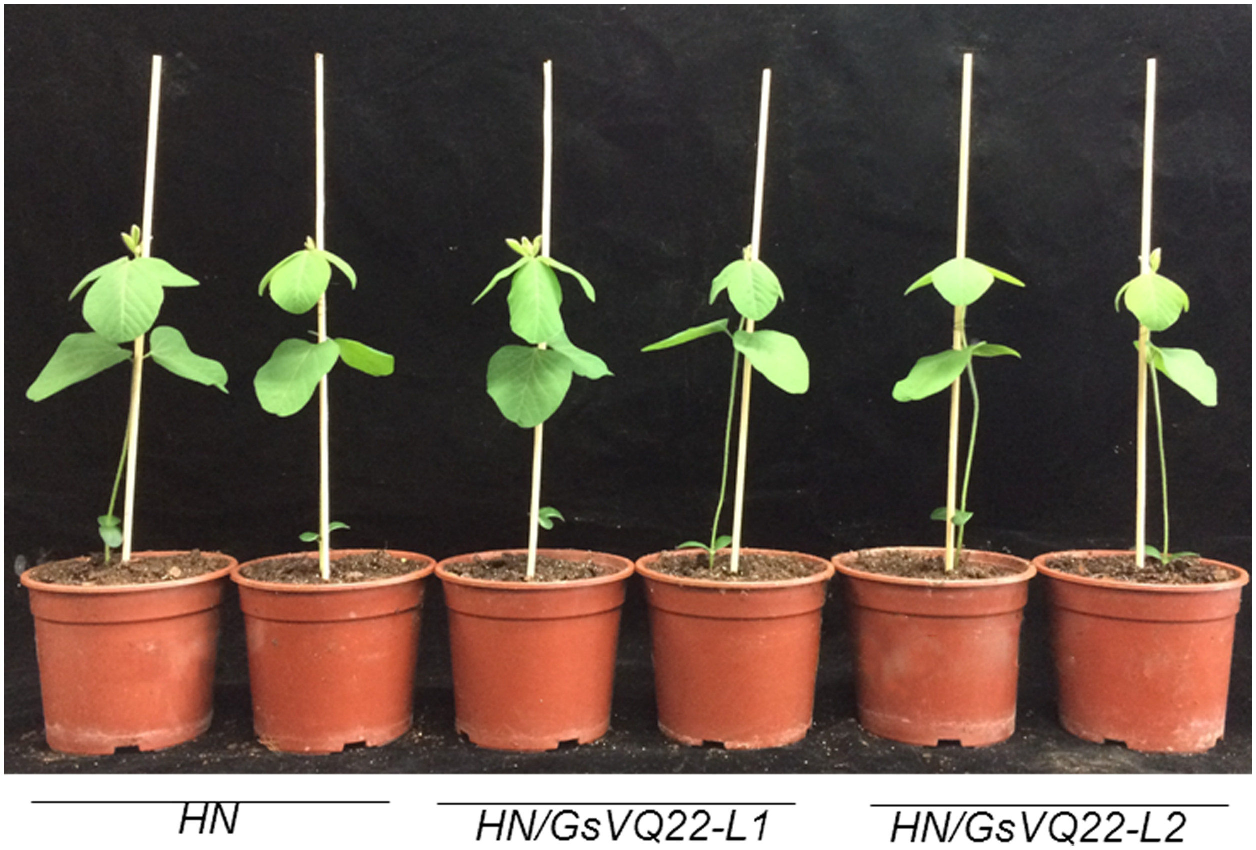

Supplemental Figure 1. Transgenic *GsVQ22* soybean plants and nontransgenic control plants at the seedling stages. Nontransgenic (HN) and two independent transgenic GsVQ22 (HN/GsVQ22-L1 and L2) soybean plants were grown in a growth room at 25°C with a 12/12 h hour light/dark photoperiod. Pictures of the plants were taken two weeks after germination.

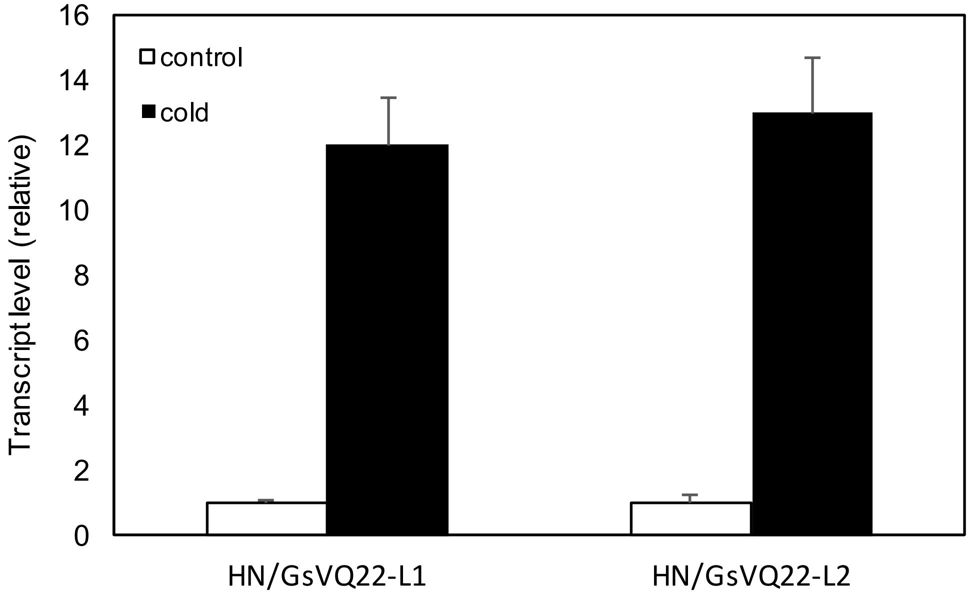

Supplemental Figure 2. qRT-PCR analysis of cold-induced expression of the *GsVQ22* transgene in transgenic soybean plants. Two independent lines (L1 and L2) of transgenic *GsVQ22* plants (HN/GsVQ22) were subjected to cold treatment (at 3°C for 48 hours). Leaf samples were collected before and after cold treatment for RNA isolation and qRT-PCR analysis of transcript levels for the *GsVQ22* transgene using gene-specific primers.

Supplemental Table 1. Correction of the Phytozome Idendifers (ID) and names of VQ proteins from *G. max*.

From Table 1 of Wang et al.[a]				Corrected	
Name	PhytozomeID	AA	VQ motif	PhytozomeID	Name
GmVQ1	Glyma01g02280.1	318	INVGRQQQQPQPQVYNISKNEFRDIVQKLTGSPSQD	Glyma01g12940.2	GmVQ2
GmVQ2	Glyma01g12940.2	289	SWKKAPVAPPAPTPIRVYKVDAINFRDLVQQLTGAPEFKP	Glyma01g40320.1	GmVQ3
GmVQ3	Glyma01g40320.1	154	RQQPQPQVYNISKNDFRDIVQQLTGSPSQD	Glyma01g02280.1	GmVQ1
GmVQ4	Glyma02g29281.1	192	PKIRIIHIFAPEIIKTDVENFRELVQKLTGRPSGENLK	Glyma02g29281.1	GmVQ4
GmVQ5	Glyma02g37135.1	212	TFNKQYALPSESQPQVYNISKNDFRDMVQKLTGSPGH	Glyma02g37135.1	GmVQ5
GmVQ6	Glyma03g27560.1	233	VKITYISNPVLVRACDASEFRSVVQQLTGKDTN	Glyma03g40941.1	GmVQ9
GmVQ7	Glyma03g28360.1	167	KISKRKSRASKRSQTTFITADPANFRQMVQQVTGVRFGGAGQ	Glyma03g27560.1	GmVQ6
GmVQ8	Glyma03g36330.1	119	VKVVYISNPMKIKTSASEFRALVQELTGQDAESPP	Glyma03g28360.1	GmVQ7
GmVQ9	Glyma03g40941.1	127	PVIVHLKSPKVIHVRPEEFMSLVQQLTRNPVSAA	Glyma03g36330.1	GmVQ8
GmVQ10	Glyma04g10780.1	287	RNSKKRSRASRRAPTTVLTTDTNNFRSMVQEFTGIPAPPFSP	Glyma04g11211.1	GmVQ11
GmVQ11	Glyma04g11211.1	313	VKVTYISSPMKVKTSASNFRALVQELTGQASN	Glyma04g16880.1	GmVQ12
GmVQ12	Glyma04g16880.1	127	QQREPVIIYSVSPKVIHVTPGDFMDVVQRLTGASSGEY	Glyma04g39270.2	GmVQ13
GmVQ13	Glyma04g39270.2	280	RQPIIIYTVSPKVIHTTPSDFMNLVQRLTGSSSSSS	Glyma04g41820.1	GmVQ14
GmVQ14	Glyma04g41820.1	261	PNNQVQAQQNLQHQPPVYNINKNDFRDVVQKLTGSPAHD	Glyma04g10780.1	GmVQ10
GmVQ15	Glyma05g22760.1	186	PWKKAPVAPMPPTPIKVYKVDAINFRDVVQQLTGAPEHES	Glyma05g22760.1	GmVQ15
GmVQ16	Glyma05g26340.1	211	KPIRRRSRTSKSTPITLLKANTSNFRALVQQFTGCPTTTTMS	Glyma05g26340.1	GmVQ16
GmVQ17	Glyma05g27220.1	113	RNPKKRSRASRRAPTTVLTTDTTNFRAMVQEFTGIPAPPFTS	Glyma05g31341.1	GmVQ18
GmVQ18	Glyma05g31341.1	486	AASDCKPLTTFVQTNSDAFREVVQRLTGPSEASAAK	Glyma05g33250.5	GmVQ20
GmVQ19	Glyma05g32350.1	208	PKIVHIETRYVETDAIHFRDVVQHLTGKN	Glyma05g27220.1	GmVQ17
GmVQ20	Glyma05g33250.5	186	EQRKPVIIYTVSPKVLHVTVSDFMNVVQRLTGPSSGAE	Glyma05g32350.1	GmVQ19
GmVQ21	Glyma06g10630.1	295	RQPIIIYTVSPKVIHTTPSDFMNLVQRLTGSSSSSSAE	Glyma06g12960.2	GmVQ24
GmVQ22	Glyma06g10951.1	341	QQQREPVIIYSVSPKVIHVTPGDFMDVVQRLTGASIGEE	Glyma06g15650.2	GmVQ25
GmVQ23	Glyma06g10960.2	316	KTIPRSDSTTNPYPTTFVQADTSTFKHVVQMLTGSSETTNP	Glyma06g36640.1	GmVQ26
GmVQ24	Glyma06g12960.2	249	PNNQVQHQPPVYNINKNDFRDVVQKLTGSPAHD	Glyma06g10630.1	GmVQ21
GmVQ25	Glyma06g15650.2	222	RNSKKRSRSSRRAPTTDTNNFRSMVQEFTGIPAPPFSS	Glyma06g10951.1	GmVQ22
GmVQ26	Glyma06g36640.1	244	RIQGNESRASRRAPTTVLTTDTNNFRSMVQEFTGISAPPFSS	Glyma06g10960.2	GmVQ23
GmVQ27	Glyma07g03240.1	193	TLHSPTTPNTTFVQANPSNFRAVVQKLTGASDDPSAH	Glyma07g03240.1	GmVQ27
GmVQ28	Glyma07g10290.2	247	QQQCHPVIIYTHSPKVIHTQPKDFMSLVQKLTGLSRSDEK	Glyma07g10290.2	GmVQ28
GmVQ29	Glyma07g31781.1	392	KPVTRSESANPYPTTFVQADTTSFKQVVQMLTGSTQTAKQ	Glyma07g31781.1	GmVQ29
GmVQ30	Glyma08g00850.1	174	KQIRRRSRASKSTPITLLKANTSNFRALVQQFTGCPTTTAMS	Glyma08g09250.1	GmVQ32

GmVQ31	Glyma08g04651.1	140	TLHSPTTPNTTFVQADPSNFRAVVQKLTGASDDPSAP	Glyma08g22860.1	GmVQ38
GmVQ32	Glyma08g09250.1	221	QQQRPPVIIYTHPPKVIHTHARNFMELVQKLTGLYRTDP	Glyma08g04651.1	GmVQ31
GmVQ33	Glyma08g10166.1	111	PKIVHIETRYVETDAINFRDVVQHLTGKN	Glyma08g10166.1	GmVQ33
mVQ34	Glyma08g14581.1	472	RNPKKRSRASRRAPTTVLTTDTTNFRAMVQEFTGIPAQPFTS	Glyma08g14581.1	GmVQ34
GmVQ35	Glyma08g15620.1	198	EHRKPVIIYAVSPKVLHVPAGDFMNVVQRLTGPSS	Glyma08g15620.1	GmVQ35
GmVQ36	Glyma08g16790.1	141	VKIVIINTQYVETDATSFKSVVQKLTGKD	Glyma08g18820.1	GmVQ37
GmVQ37	Glyma08g18820.1	89	TLIKVLRPKVYITDSSSFKKLVQELTGNGSSN	Glyma08g42125.1	GmVQ42
GmVQ38	Glyma08g22860.1	194	AAASDCKPLTTFVQTNSDAFREVVQRLTGPSEASAAK	Glyma08g00850.1	GmVQ30
GmVQ39	Glyma08g36730.1	292	MNGGRQQQQQPQPQVYNISKNDFRDIVQQLTGSPSQSQDH	Glyma08g36730.1	GmVQ39
GmVQ40	Glyma08g36740.1	294	MNGGRQQQQQPQPQVYNISKNDFRDIVQQLTGSPSQSQDH	Glyma08g36750.1	GmVQ41
GmVQ41	Glyma08g36750.1	292	MNGGRQQQQQPQPQPQVYNISKNDFRDIVQQLTGSPSQSQDH	Glyma08g36740.1	GmVQ40
GmVQ42	Glyma08g42125.1	182	PKIRIIHVYTPEIIKTDAANFRELVQRLTGKP	Glyma08g16790.1	GmVQ36
GmVQ43	Glyma09g05700.1	244	KPIRRRSRASKRTPTTLLNANPTNFRALVQQFTGCPRTTMST	Glyma09g05700.1	GmVQ43
GmVQ44	Glyma09g17151.1	203	PKIRIIHIFAPEIIKTDVENFRELVQKLTGKPSGENLK	Glyma09g17151.1	GmVQ44
GmVQ45	Glyma09g31600.2	243	QQHHHPVIIYTHSPKVIHTQPKDFMSLVQKLTGLSRSGE	Glyma09g31600.2	GmVQ45
GmVQ46	Glyma10g41970.2	191	PQRINPIIIYTESPKIIHTKAKDFMALVQRLTGRSSST	Glyma10g41970.2	GmVQ46
GmVQ47	Glyma11g04970.1	155	PWKKAAVAPPAPTPIRVYKVDAINFRDLVQQLTGAPEFKPA	Glyma11g04970.1	GmVQ47
GmVQ48	Glyma11g38180.1	439	RNPKKRSRASRRAPTTVLTTDTTNFRAMVQEFTGIPAPPFTS	Glyma11g38180.1	GmVQ48
GmVQ49	Glyma12g24250.1	248	KTILRSDSTNPYPTTFVQADTSTFKQVVQMLTGSSETTKPQ	Glyma12g24250.1	GmVQ49
GmVQ50	Glyma12g35380.1	246	KTIPRSDSNPYPTTFVQADTSTFKQVVQMLTGSSDTTKQ	Glyma12g35380.1	GmVQ50
GmVQ51	Glyma13g08831.1	240	KPMTRSEPANSYPTTFVQADTTSFKQVVQMLTGSSETAKQ	Glyma13g31180.1	GmVQ56
GmVQ52	Glyma13g10840.1	224	KTIPRSDSNPYPTTFVQADTSTFKQVVQMLTGSSDTTKQ	Glyma13g35130.1	GmVQ57
GmVQ53	Glyma13g24700.1	281	KSVTRSESGNPYPTTFVQADTNSFKQVVQMLTGSTQTAKQ	Glyma13g24700.1	GmVQ53
GmVQ54	Glyma13g26290.3	119	MEAPKIVQIETRYVQTDAANFRDVVQSLTGKNSSTDWI	Glyma13g29005.1	GmVQ55
GmVQ55	Glyma13g29005.1	100	RQPVIIYTVSPKVIHTTPSDFMSLVQRLTSSSSSSS	Glyma13g08831.1	GmVQ51
GmVQ56	Glyma13g31180.1	208	IKVTYISSPVKVKTSASNFRALVQELTGQYSN	Glyma13g26290.3	GmVQ54
GmVQ57	Glyma13g35130.1	249	KPTRRRSRASRRTPTTLLNTDTTNFRAMVQQFTGGPSAPFAS	Glyma13g10840.1	GmVQ52
GmVQ58	Glyma14g00570.1	161	KPIRRRSRASRRTPTTLLNTDTSNFRAMVQQFTGAPSAP	Glyma14g00570.1	GmVQ58
GmVQ59	Glyma14g29580.2	237	RQPVIIYTVSPKVIHTTPSDFMSLVQRLTGSSSSSS	Glyma14g29580.2	GmVQ59
GmVQ60	Glyma14g34681.1	429	RNSKKRTRASRRAPTTVLTTDTSNFRAMVQEFTGIPAPPFSA	Glyma14g34681.1	GmVQ60
GmVQ61	Glyma15g08160.1	199	MTRSEPANPYPTTFVQADTSSFKQVVQMLTGSSETAKQ	Glyma15g08160.1	GmVQ61
GmVQ62	Glyma15g16990.1	252	KPIRRRSRASKKTPTTLLNANTTNFRALVQQFTGCHSTTMPT	Glyma15g16990.1	GmVQ62
GmVQ63	Glyma15g37230.1	122	PKIRIIHVYAPEIIKTDAANFKELVQRLTGKP	Glyma15g42280.2	GmVQ65
GmVQ64	Glyma15g40000.1	89	IKVTYISSPVKVKTSASNFRALVQELTGQYSN	Glyma15g37230.1	GmVQ63
GmVQ65	Glyma15g42280.2	157	VKVVIINTQYVETDATSFKSVVQKLTGKD	Glyma15g40000.1	GmVQ64

GmVQ66	Glyma17g17210.1	190	PWKKHHVAPMPPTPVKVYKVDAINFREVVQQLTGA PKHKP	Glyma17g17210.1	GmVQ66
GmVQ67	Glyma18g02140.1	454	RNPKKRSRASRRAPTTVLTTDTTNFRAMVQEFTGIP APPFTS	Glyma18g02140.1	GmVQ67
GmVQ68	Glyma18g13001.1	179	TLIKILRPKVYITDSSSFKKLVQELTGNGSP	Glyma18g13001.1	GmVQ68
GmVQ69	Glyma19g30531.1	232	VKITYISSPVLVRAYDASEFRSVVQQLTGNDSNS	Glyma19g43590.1	GmVQ72
GmVQ70	Glyma19g31080.1	168	VKVVYISNPMKIKTSASEFRALVQELTGQDAESPP	Glyma19g31080.1	GmVQ70
GmVQ71	Glyma19g38980.1	124	PVIVHLKSPKVIHVRPEEFMSLVQQLTGNPVSAA	Glyma19g38980.1	GmVQ71
GmVQ72	Glyma19g43590.1	102	KISKRKSRASKRSQTTFITADPANFRQMVQQVTGV RFGGA	Glyma19g30531.1	GmVQ69
GmVQ73	Glyma20g15230.1	233	KPTRRRSRASRRTPTTLLNTDTTNFRAMVQQFTGG PSAPYAS	Glyma20g15230.1	GmVQ73
GmVQ74	Glyma20g25070.2	157	PQRINPIIIYTESPKVIHTKAKDFMALVQRLTGRSST	Glyma20g25070.2	GmVQ74

[a]Wang, X., Zhang, H., Sun, G., Jin, Y., and Qiu, L. (2014) Identification of active VQ motif-containing genes and the expression patterns under low nitrogen treatment in soybean. Gene, 543:237-243

Supplemental Table 2. Primers used in qRT-PCR analysis of soybean VQ genes

Gene name	Gene identifier	Primers
GmVQ1	Glyma01g02280.1	F:TACAACCGAGTCACCCATCT<br>R:GGAGGATTAGGAAGCAAAGC
GmVQ2	Glyma01g12940.2	F:ATTTCCCTTTCTCACCCTTG<br>R:CCACCTAGGACTTGTGATGG
GmVQ3	Glyma01g40320.1	F:GCACAATCTGAAGCATTGGA<br>R:GAGAACTCGACCCGTTTCC
GmVQ4	Glyma02g29281.1	F:AACTGGGAGACCAAGTGGAG<br>R:CCAACAACACCCACCATTAT
GmVQ5	Glyma02g37135.1	F:GCCTCACTCACAACAACACCT<br>R:TGGACCATGTCCCTGAAATCG
GmVQ6	Glyma03g27560.1	F:GAGAACGTAGCCAGCATTGA<br>R:GTCTGACTTGGGCAGAAAGG
GmVQ7	Glyma03g28360.1	F:AGATCAAGACCAGCGCATCC<br>R:GGGTGAATTAGGCCGTGGAA
GmVQ8	Glyma03g36330.1	F:CAAGAGTAGCACCGTGGTTG<br>R:AAAGTGCATTAAAGCTTGGAAGT
GmVQ9	Glyma03g40941.1	F:CCACAAGGAAGCCAATACCT<br>R:TGAGTTGTTGAACCACCGAT
GmVQ10	Glyma04g10780.1	F:AGCAGAACCTGCAACATCAG<br>R:GGTGTTGAGATACGGTCGTG
GmVQ11	Glyma04g11211.1	F:GCCAAAGATCTGAACCCAAT<br>R:TCTTCGAGTTGCGTATGAGG
GmVQ12	Glyma04g16880.1	F:CCTCCAATGTTGCTGAAATG<br>R:GACCTCGAATTCAAGTGGCT
GmVQ13	Glyma04g39270.2	F:GTCGGAGAGAGAAAGGGTTT<br>R:CCTACTTCCACTCCCTCCTC
GmVQ14	Glyma04g41820.1	F:GCGCCAACCCATAATAATCT<br>R:GTTGTTGGGCACCACTACAG
GmVQ15	Glyma05g22760.1	F:ATAATGATGGAGCAATGGCA<br>R:CTGGTTCCAAACTGGTGACA
GmVQ16	Glyma05g26340.1	F:CAAACCAATCCGAAGGAGAT<br>R:ATTGCAAGTGACATGGTGGT
GmVQ17	Glyma05g27220.1	F:CGTGGCATGTTCTTCAGATT<br>R:CATGGGAGGCATATCAGACA
GmVQ18	Glyma05g31341.1	F:CCTACCTTGCCTCCAGAAAG<br>R:ACCACGTTGCAGTTGTTGTT

GmVQ19	Glyma05g32350.1	F:TACACCGTGTCTCCCAAAGT<br>R:AACGTCACGTCGTTGTCTTC
GmVQ20	Glyma05g33250.5	F:TATGGTGCAGCATCAGATTG<br>R:CTTCCGAGGGACCTGTTAAA
GmVQ21	Glyma06g10630.1	F:ACCTATCTCCGCCTACATGC<br>R:CTGCTGTGGCTGTTGTTCTT
GmVQ22	Glyma06g10951.1	F:TCTGCTGGATCATGGGAATA<br>R:AAGATCAAATCCACGCTTCC
GmVQ23	Glyma06g10960.2	F:CACCAACAACAACGAATCAA<br>R:TTGAAGCTCTGCTTTCGTTT
GmVQ25	Glyma06g15650.2	F:GCGGGAACCAGTGATAATCT<br>R:TCTCTCCGAAGGACTCGTCT
GmVQ26	Glyma06g36640.1	F:TCTCCAAGCTTGCTTGACTT<br>R:GAAGAAGTGCTTCCCAATGA
GmVQ27	Glyma07g03240.1	F:AGCAGAACAACGACGACAAC<br>R:CTCCGCTATCGCTCTCTCTT
GmVQ28	Glyma07g10290.2	F:TCGGTCATAACAACCGAAGA<br>R:CTGACTCGAACACGCAAACT
GmVQ29	Glyma07g31781.1	F:CTTCAAGCAAGTCGTCCAAA<br>R:TTGAGAGGGTTGATGCTGAG
GmVQ30	Glyma08g00850.1	F:CCCTTCAACACTGTTCTCTCAG<br>R:CATTACGAGATGCCAGAGGA
GmVQ31	Glyma08g04651.1	F:TCATCTTCATCCCTGAGCAA<br>R:CGATAGAGTCCGGTGAGCTT
GmVQ32	Glyma08g09250.1	F:AGGGTTGTGCATTCAAACAA<br>R:AGTGACATGGCTGTTGTGGT
GmVQ34	Glyma08g14581.1	F:CAACCAACCTCAACCACAAC<br>R:GGTTTGGTTCGGATCTCACT
GmVQ35	Glyma08g15620.1	F:CGAAGACAACGACGTGAAGT<br>R:GAAGCTGTTCGTTGACCAAA
GmVQ36	Glyma08g16790.1	F:TGATCATGCAAGATGAAGAGG<br>R:GAACCCATCAAGGAAACCAC
GmVQ37	Glyma08g18820.1	F:GCACCCGTGAAGATTGTG<br>R:CGAACTCCTTAAACGACACG
GmVQ38	Glyma08g22860.1	F:AAAGAAGCCGAACTTCAAGC<br>R:TCCAACGTTGTTGGTGTTCT
GmVQ39	Glyma08g36730.1	F:TCGTCATCCAGCTTCTTCTG<br>R:AGGAGGTCCATTTGGTTGTG

GmVQ40	Glyma08g36740.1	F:ACAACAACCATCACCAAACC<br>R:GGCATTCAAAGGCAACATAG
GmVQ41	Glyma08g36750.1	F:GGCTTTCAGTTTCCATCTCC<br>R:AGCCTGAAGGAGACAAAGGA
GmVQ43	Glyma09g05700.1	F:CCTAATTGGCAGAAGCAAGA<br>R:TGAGTTACCCGAACTTGGAA
GmVQ44	Glyma09g17151.1	F:TGGAGAACTTCAGGGAGCTT<br>R:AATCAGAGAACCCACCCAAG
GmVQ45	Glyma09g31600.2	F:CATCATCGTCATCGTCATCA<br>R:TGTGGATGACCTTTGGAGAA
GmVQ46	Glyma10g41970.2	F:CATCATTTACACCGAATCGC<br>R:TTGAGGAAGTGAAGCAGTGG
GmVQ47	Glyma11g04970.1	F:CGTCGACCAAGCCTATTTCT<br>R:TGATCAGCTGGCTTGAACTC
GmVQ48	Glyma11g38180.1	F:ACCAAATCCTCAACCTCGAC<br>R:GGGAGAGTGGTACTGGGAAA
GmVQ49	Glyma12g24250.1	F:ACATTCCTCCAATGAAAGCA<br>R:TTCTTGAGGTGGTGGTTGTT
GmVQ50	Glyma12g35380.1	F:TCTGACTCCAACCCTTACCC<br>R:AGCCTTGTTGCTTCTTTGGT
GmVQ52	Glyma13g10840.1	F:ATCAGGCTCGACCCATTTA<br>R:TTGTGGTGTCTGTGTTGAGG
GmVQ53	Glyma13g24700.1	F:ATCAACCCTCTCATCGGAAC<br>R:GGGAGTAACAGGGCTGAGAA
GmVQ54	Glyma13g26290.3	F:GCTGAAACCTCCATGCCTAT<br>R:TTGTTGTTGGTTGAATGGCT
GmVQ56	Glyma13g31180.1	F:ACACAACCTCGTTCAAGCAA<br>R:CTGCTGCTTCTTGTTGGGTA
GmVQ57	Glyma13g35130.1	F:CTGCTGCTTCTTGTTGGGTA<br>R:GGTTGTGGATCTTGTGATGC
GmVQ58	Glyma14g00570.1	F:TGATCACTGGCTCCATTTCT<br>R:GAGTGGTGGGAGTTCTCCTT
GmVQ59	Glyma14g29580.2	F:TCCGAAAGTAATCCACACCA<br>R:TCACTACCACTGCCACCATT
GmVQ60	Glyma14g34681.1	F:CATTCCTCAACTTGGACACC<br>R:GAGAACCCAACAAACATTGG
GmVQ61	Glyma15g08160.1	F:CATGATGACGAGATCCGAAC<br>R:GGATCGAGTTCGAAGGTTTC

GmVQ62	Glyma15g16990.1	F:ACCATTTAGTGGCAGAAGCC<br>R:GTAACCACTCTGCTGAGGCA
GmVQ63	Glyma15g37230.1	F:TCCAATGTTGCTGAAACCTC<br>R:TTGTTGTTGGTTGAATGGCT
GmVQ64	Glyma15g40000.1	F:AAGAGGCATGATCATAAAGGTG<br>R:GTCGTTTAACGGTGGCATC
GmVQ65	Glyma15g42280.2	F:CTGCAAATTTCAAAGAGTTGG<br>R:GAATCCTTTGGGTCTTTGGT
GmVQ66	Glyma17g17210.1	F:AACAACCGTGAGGACTCAAA<br>R:TGCTGAACAACTTCACGAAA
GmVQ67	Glyma18g02140.1	F:GCCACTGCGACGTTAAACTA<br>R:AGGATTCGACCATACCTTCG
GmVQ70	Glyma19g31080.1	F:TCACCGTGTGAATTGTGTTG<br>R:CAGGGCAGAGATGTTCTCAA
GmVQ71	Glyma19g38980.1	F:TAAGCAGAAGCAGCACCTCT<br>R:TGCTGCACGAGACTCATAAA
GmVQ72	Glyma19g43590.1	F:GCAATGACTCCAACTCCAAC<br>R:AACGTGCAAGTTCTTTCCAG
GmVQ73	Glyma20g15230.1	F:CATATGCATCAAACCCATCA<br>R:GCCACCACCACCAGTACTAT
GmVQ74	Glyma20g25070.2	F:ACACCAAGGCCAAAGATTTC<br>R:CCCTCTTGAGGAAGTGAAGC

Supplemental Table 3. Primers used for generating pAD-VQ fusion constructs.

Gene name	Gene identifier	Primers
GmVQ1	Glyma01g02280.1	F:AGCGCTAGCATGGATAACTCCAAAAACAGGCA<br>R:AGCCTCGAGAGCACTAGTGCTCTCTCCACCTGGGA
GmVQ2	Glyma01g12940.2	F:AGCGAATTCATGGATCACAAACACTGTCAAACT<br>R:AGCCTCGAGCTACATCAATCTCATATCGCGAGA
GmVQ3	Glyma01g40320.1	F:AGCGAATTCATGGACACCATTCGCAGTAC<br>R:AGCCTCGAGTCACCAGTGATTAAGATGAGGGGA
GmVQ4	Glyma02g29281.1	F:AGCGGATCCATGGATTCCCAAGCAACAAGC<br>R:AGCCTCGAGTTAAAAAAAATCTACAAAGTGCATTAAAG<br>C
GmVQ5	Glyma02g37135.1	F:AGCGAATTCATGGGAAAACTGAGGTATGATC<br>R:AGCCTCGAGCTAATGTTGCATGACCCTTTGTT
GmVQ6	Glyma03g27560.1	F:AGCGAATTCAGCCCATGGATCCGCCGGAGATTC<br>R:AGCCTCGAGTTACAAGTCGAAGAGACTGAAGAGG
GmVQ7	Glyma03g28360.1	F:AGCGAATTCATGAACGATAACATGACCGC<br>R:AGCCTCGAGTCACATAAATAAACCATCGCTATCA
GmVQ8	Glyma03g36330.1	F:AGCGGATCCATGGACCCGCCGGAATTCC<br>R:AGCCTCGAGTTAGTAAAAGAGATTAAAGATGTCCG
GmVQ9	Glyma03g40941.1	F:AGCGAATTCATGGGAATGGAGAAGCCACC<br>R:AGCCTCGAGTTATACTTTCTCACTTGCATTAGGAGA
GmVQ22	Glyma06g10951.1	F:AGCCTCGAGATGGACTCTGGTAACAGTGGA<br>R:AGCTCTAGATCACACCCCACGCGAGATCATATT
GmVQ26	Glyma06g36640.1	F:AGCGAATTCATGGAAATCAACTTAAGGCTCCA<br>R:AGCCTCGAGTCAAGTAGTTGACTCAGAAACTCTAGG
GmVQ27	Glyma07g03240.1	F:AGCGAATTCATGGCTGCTTCCAACTCG<br>R:AGCTCTAGATTAATTAGGAATTGATGGTGAATTG
GmVQ28	Glyma07g10290.2	F:AGCGAATTCATGGCAATGTCATTATACAAACA<br>R:AGCCTCGAGTCAATGCTCACGGAACTCTTT
GmVQ35	Glyma08g15620.1	F:AGCGGATCCATGGACCCGCCGGAATTTC<br>R:AGCCTCGAGTTAGTCGAAGAGATTAAAGATGTCC
GmVQ43	Glyma09g05700.1	F:AGCGAATTCATGAGGGATAACATGACCATGACT<br>R:AGCCTCGAGCTATAATTCACTCTCATAAAGAGCATCAT
GmVQ44	Glyma09g17151.1	F:AGCGGATCCATGCACAAAGGTTCCCAC<br>R:AGCCTCGAGTCATGTGATTCCCATCCA
GmVQ52	Glyma13g10840.1	F:AGCGAATTCATGACCCAAACCATGTCAGGC<br>R:AGCCTCGAGTCAAGAAGAAGAAGAAGTGTTTGG
GmVQ53	Glyma13g24700.1	F:AGCGAATTCATGGACTCTCCGAGGTACCA<br>R:AGCCTCGAGTTAAGAGGAGGAATTAGAAGAAGCA

GmVQ59	Glyma14g29580.2	F:AGCGAATTCATGATTAACAAGGACTCCCACAA<br>R:AGCCTCGAGTCAAGGTGAAACAAAGCTTGAA
GmVQ62	Glyma15g16990.1	F:AGCGAATTCATGAGGGATATGACCATGGC<br>R:AGCCTCGAGCTATAATTCACTCTTATAAAAGGTATCATTGG
GmVQ63	Glyma15g37230.1	F:AGCGAATTCATGAAGAACAAAAGGGCAAGC<br>R:AGCCTCGAGCTATGACATGTCAAAACTCATCAAATC
GmVQ64	Glyma15g40000.1	F:AGCGAATTCATGTCTACCAGTGGATGCACC<br>R:AGCCTCGAGTTAATCAGCCCAAAGGTCGT
GmVQ66	Glyma17g17210.1	F:AGCGGATCCATGGAAGCCTATTCTGGTTCTT<br>R:AGCCTCGAGGAACCTTCCCCGGTTCCAA
GmVQ67	Glyma18g02140.1	F:AGCGCTAGCATGGATTCGGGCAACAGT<br>R:AGCCTCGAGTAATCAGAAGAACAATTAATCCAGG
GmVQ74	Glyma20g25070.2	F:AGCGGATCCATCAATGGTCCACGTCCTTC<br>R:AGCCTCGAGTCAATATTCAGGCAACTCTTTCA

Supplemental Table 4. Primers used for generating pBD-WRKY fusion constructs.

Gene name	Gene identifier	Primers
GMWRKY9	Glyma01g06550.1	F:AGCGAATTCGATCCAGCTTCTTCACATAGGACT<br>R:AGCGTCGACAACTGTGATGCAGTATTGCTGG
GMWRKY28	Glyma01g06870.1	F:AGCGAATTCGGTAAAAACAAGGGGCAAAAGC<br>R:AGCGTCGACCTGGCTGGCAAAGGCAGCTT
GMWRKY54	Glyma02g01420.1	F:AGCGAATTCCCTAAGAAGACAAATCAGAAGAG<br>R:AGCGTCGACTGAACCGAAGTTGTTGGCGT
GMWRKY96	Glyma02g36510.1	F:AGCGAATTCGAAATGGATTCTCCAGTAAAACC<br>R:AGCGTCGACTGCAGCAGCCACAAGAGGA
GMWRKY49	Glyma02g39870.1	F:AGCGAATTCATGTCTGCCCCTGGAAGTA<br>R:AGCGTCGACATTGTTTGGCATTGGTCTGTTAAC
GMWRKY3	Glyma02g46690.1	F:AGCGAATTCGACATCACTCCAGTAGTTAAGC<br>R:AGCGTCGACACCTGCTGTAGCTGCTGGT
GMWRKY141	Glyma03g25770.1	F:AGCGAATTCGGAAATACTAGTGATGGTGGCA<br>R:AGCGTCGACAAAAGAGGTGAAACATTCATGTTCCGA
GMWRKY51	Glyma03g37940.1	F:AGCGAATTCGCTAAGAAGACAAATCAGAAGAG<br>R:AGCGTCGACAGCAGATCCAGACATAATTACCGA
GMWRKY93	Glyma04g12830.1	F:AGCGAATTCGAGTTGAGTGGAGCTACTAGA<br>R:AGCGTCGACAGGAACTGCATTGGAAGCAT
GMWRKY142	Glyma04g39620.1	F:AGCGAATTCAAGAAAATGAAAGCAAGGAGAAAGGT<br>R:AGCGTCGACCCACAAGAAATTACTAAGTTCAGA
GMWRKY130	Glyma05g25770.1	F:AGCGAATTCGAGAAGAAGAAAGGAGAGAAGA<br>R:AGCGTCGACCAAAGAAGAAGGTGTGAACATTCC
GMWRKY115	Glyma05g31800.1	F:AGCGAATTCAATGGGATTAATGAAAACAAAAGAGG<br>R:AGCGTCGACGAAAGTAGTATCAGAATGTACGAAGG
GMWRKY101	Glyma07g36640.1	F:AGCGAATTCCCAAAAAAGAAGAACCAAAAGAAGC<br>R:AGCGTCGACCCCACCACTTGCTTCGGAATGCA
GMWRKY4	Glyma08g26230.1	F:AGCGAATTCGAAGTACCTCTGTCTCAGAAGA<br>R:AGCGTCGACTAATGGCATTGAATTGCTACTAGCTG
GMWRKY91	Glyma16g05880.1	F:AGCGAATTCAATAAAAAGAAAGGAGAGAAGAAGGT<br>R:AGCGTCGACAGTGTATATTTTCATCTGACTCAAAATG
GMWRKY135	Glyma04g05700.1	F:AGCGAATTCGTTGGCAATGAACGTGAGAA<br>R:AGCGTCGACGATATAACTCTGGTGATTGTGGA
GMWRKY81	Glyma06g27440.1	F:AGCGAATTCGATTTGGATACGGCAGTAAAAC<br>R:AGCGTCGACTGCCGCAGCCACAAGTGAA
GMWRKY102	Glyma07g35380.1	F:AGCGAATTCGATCCAGCCTCTTTACATAGA<br>R:AGCGTCGACTAGCTGTGATGCACTATTGTTGG

GMWRKY25	Glyma08g01430.1	F:AGCGAATTCCCAAGCCTGAAAGGTGGCAA<br>R:AGCGTCGACGCTGTATATGTGATGGTTTCTCAA
GMWRKY19	Glyma11g29720.1	F:AGCGAATTCATATCAGCTGTAGGAAGTAGAACA<br>R:AGCGTCGACTATTATTGGCAATGATCTACTGATAGAG
GMWRKY129	Glyma05g25270.1	F:AGCGAATTCACTACCCATGCTGCTTCCCCTC<br>R:AGCGTCGACGAAGCTGCTGCAGAAGCTGTGG
GMWRKY30	Glyma13g36540.1	F:AGCGAATTCATGAAGAAGAGGGTGGTGAC<br>R:AGCGTCGACGGCGCCGTCGCTTACAGCGG
GMWRKY161	Glyma18g44560.1	F:AGCGAATTCATTAAGGAACGTAGAGGGTG<br>R:AGCGTCGACCTTGGATGAGGATGAAGGGG

Supplemental Table 5. Primers for generating GmVQ gene expression constructs.

Gene name	Gene identifier	Primers
GmVQ1	Glyma01g02280.1	F:AGCCCATGGATAACTCCAAAAACAGGCA<br>R:AGCACTAGTGCTCTCTCCACCTGGGA
GmVQ2	Glyma01g12940.2	F:AGCCCATGGATAACTCAAAAAACAGGCA<br>R:AGCTCTAGATCACCACCTAGGACTTGTGAT
GmVQ3	Glyma01g40320.1	F:AGCCATGGAAGCTTATTCTGGTTCTTAC<br>R:AGCTCTAGAGAACTCGACCCGTTTC
GmVQ4	Glyma02g29281.1	F:AGCCTCGAGATGCACAAAGATTCCCACA<br>R:AGCTCTAGATCAAAAATCAGAAATGAACCCTTC
GmVQ5	Glyma02g37135.1	F:AGCCCATGGATCACAAACACTGTCAAACT<br>R:AGCTCTAGACTACATCAATCTCATATCGCGAGA
GmVQ6	Glyma03g27560.1	F:AGCCCATGGCATCATCCGAGAACGT<br>R:AGCTCTAGATCACATGACTTTCCACGACTC
GmVQ7	Glyma03g28360.1	F:AGCCCATGGACACCATTCGCAGTAC<br>R:AGCTCTAGATCACCAGTGATTAAGATGAGGGGA
GmVQ8	Glyma03g36330.1	F:AGCCCATGGATTCCCAAGCAACAAGC<br>R:AGCTCTAGATTAAAAAAAATCTACAAAGTGCATTAAAGC
GmVQ9	Glyma03g40941.1	F:AGCCCATGGGAAAACTGAGGTATGATC<br>R:AGCTCTAGACTAATGTTGCATGACCCTTTGTT
GmVQ10	Glyma04g10780.1	F:AGCCCATGGATAAAAGCTGTGATCCCT<br>R:AGCTCTAGATCAAATTCCCTTCCAGCGAGGGCT
GmVQ11	Glyma04g11211.1	F:AGCCTCGAGATGCAATGGATTCTGGCAAC<br>R:AGCTCTAGACTATTGATCAGAAGAACAAATCCA
GmVQ12	Glyma04g16880.1	F:AGCCTCGAGATGCTTGGCGTTAATAACAAC<br>R:AGCTCTAGACTACTGCATACCAAAGCTCAAAAG
GmVQ13	Glyma04g39270.2	F:AGCCCATGGATCCGCCGGAGATTC<br>R:AGCTCTAGATTACAAGTCGAAGAGACTGAAGAGG
GmVQ14	Glyma04g41820.1	F:AGCCCATGGATCAATTCTCACACATTATTC<br>R:AGCTCTAGACTAATCTAAGAAATAGTTGAATAAGTCCATAG
GmVQ15	Glyma05g22760.1	F:AGCCCATGGAAGCCTATTCTAGTTCTTATTCC<br>R:AGCTCTAGAGAACCTTCCCTGGTTCC
GmVQ16	Glyma05g26340.1	F:AGCCTCGAGATGAACGATAACATGACCGC<br>R:AGCACTAGTTCACATAAATAAACCATCGCTATCA
GmVQ17	Glyma05g27220.1	F:AGCCTCGAGATGACGAGTGGACCAAAGATAG<br>R:AGCTCTAGACTACAACATGAGCAACTCCTCCA
GmVQ18	Glyma05g31341.1	F:AGCCTCGAGATGGATTCGGCCAACACT<br>R:TCAGTGAAGACTTCGAAGTTGGAGT

GmVQ19	Glyma05g32350.1	F:AGCCCATGGACCCGCCGGAATTCC<br>R:AGCTCTAGATTAGTAAAAGAGATTAAAGATGTCCG
GmVQ20	Glyma05g33250.5	F:AGCCCATGGGAATGGAGAAGCCACC<br>R:AGCTCTAGATTATACTTTCTCACTTGCATTAGGAGA
GmVQ21	Glyma06g10630.1	F:AGCCCATGGATAAAAGCTGTCATCCCT<br>R:AGCTCTAGATCAAATTCCCTTCCAGCGAGGGCTA
GmVQ22	Glyma06g10951.1	F:AGCCTCGAGATGGACTCTGGTAACAGTGGA<br>R:AGCTCTAGATCACACCCCACGCGAGATCATATT
GmVQ23	Glyma06g10960.2	F:AGCGTCGACATGGATTCTATCAACAGTGGAA<br>R:AGCTCTAGACTATTGCTCAGAGGAACAAATCCACG
GmVQ25	Glyma06g15650.2	F:AGCCCATGGGTTCCAAGCTCCGTT<br>R:AGCTCTAGATTACAAGTCGAAGAGGCTGAAC
GmVQ26	Glyma06g36640.1	F:AGCCCATGGAAATCAACTTAAGGCTCCA<br>R:AGCTCTAGATCAAGTAGTTGACTCAGAAACTCTAGG
GmVQ27	Glyma07g03240.1	F:AGCCCATGGCTGCTTCCAACTCG<br>R:AGCTCTAGATTAATTAGGAATTGATGGTGAATTG
GmVQ28	Glyma07g10290.2	F:AGCCCATGGCAATGTCATTATACAAACA<br>R:AGCTCTAGATCAATGCTCACGGAACTCTTT
GmVQ29	Glyma07g31781.1	F:AGCCCATGGACTCTCCGAGGTACCA<br>R:AGCTCTAGATTAAGAGGAGGAAGAAGAACCGGA
GmVQ30	Glyma08g00850.1	F:AGCCCATGGAGAAGCCACCAATCC<br>R:AGCTCTAGATTATACTTTCTCACTTGCATTACGA
GmVQ31	Glyma08g04651.1	F:AGCCTCGAGATGAACAGGGAATCTCATCTG<br>R:AGCTCTAGATCACTCCACCTCCTCTGG
GmVQ32	Glyma08g09250.1	F:AGCCTCGAGATGACTGCCACAGCTGATCA<br>R:AGCACTAGTCACATAAATAAATCATCGATATCATTGGAG
GmVQ34	Glyma08g14581.1	F:AGCCCATGGATTCGGCAAACACCA<br>R:AGCTCTAGATTAATCAGAAGAACAATTAATCCAGGAC
GmVQ35	Glyma08g15620.1	F:AGCCCATGGACCCGCCGGAATTTC<br>R:AGCTCTAGATTAGTCGAAGAGATTAAAGATGTCC
GmVQ36	Glyma08g16790.1	F:AGCCTCGAGATGACAAAGCTAGGAATTAACAAAG<br>R:AGCTCTAGATTAATTTTGATTCCCTGCAGGCAAAGTACTTAACT CT
GmVQ37	Glyma08g18820.1	F:AGCCTCGAGATGTCTAGTGGTGGATGCAGC<br>R:AGCTCTAGATTAATCAGCCCAAAGGTCGT
GmVQ38	Glyma08g22860.1	F:AGCCCATGGCTGCTTCTGCTTCAAA<br>R:AGCTCTAGATTAGGAATTGGTGGTGTTGTTG
GmVQ39	Glyma08g36730.1	F:AGCCCATGGATGAGTCAAAAAACAGGC<br>R:AGCTCTAGATCACCACCTAGGACTTGTCATGGGGA

GmVQ40	Glyma08g36740.1	F:AGCCCATGGATGAGTCAAAAAACAGGC<br>R:AGCTCTAGATCACCACCTAGGACTTGTCATGGGGA
GmVQ41	Glyma08g36750.1	F:AGCCCATGGATGAGTCAAAAAACAGGC<br>R:AGCTCTAGATCATGTTACACTCCATTCATCCA
GmVQ43	Glyma09g05700.1	F:AGCCTCGAGATGAGGGATAACATGACCATGACT<br>R:AGCACTAGTCTATAATTCACTCTCATAAAGAGCATCAT
GmVQ44	Glyma09g17151.1	F:AGCCTCGAGATGCACAAAGGTTCCCACA<br>R:AGCTCTAGATCATGTGATTCCCATCCAAA
GmVQ45	Glyma09g31600.2	F:AGCCTCGAGGAATCTCATTTCATCAAAAAATCA<br>R:AGCTCTAGATCAATGCTCAAGGAACTCTTTC
GmVQ46	Glyma10g41970.2	F:AGCCTCGAGATGATCAACAAAGATTCTCACATG<br>R:AGCTCTAGATCAATATTCAGGTAGCTCTTTCATGAATTC
GmVQ47	Glyma11g04970.1	F:AGCCCATGGAAGCTTATTATTCCGGTTCT<br>R:AGCTCTAGACTAGAGAACCCTCCCCGGTTCCAAGCT
GmVQ48	Glyma11g38180.1	F:AGCCTCGAGATGGATTCGGGTAACAGTGG<br>R:AGCTCTAGATTACCAAATAGAGACACAAATTACGA
GmVQ49	Glyma12g24250.1	F:AGCCCATGGAAATCAGCTTAAGGCTC<br>R:AGCTCTAGATCAAGAAGGTGACTCAGAAACTCT
GmVQ50	Glyma12g35380.1	F:AGCCCATGGAAATCAGCTCAAGGCT<br>R:AGCTCTAGATCAAGAGGGTGATTCTGATGA
GmVQ52	Glyma13g10840.1	F:AGCCTCGAGATGACCCAAACCATGTCAGGC<br>R:AGCTCTAGATCAAGAAGAAGAAGAAGTGTTTGG
GmVQ53	Glyma13g24700.1	F:AGCCCATGGACTCTCCGAGGTACCA<br>R:AGCTCTAGATTAAGAGGAGGAATTAGAAGAAGCA
GmVQ54	Glyma13g26290.3	F:AGCCTCGAGATGCCGACAGTGGCGGTG<br>R:AGCTCTAGACTATGACATGTCAAAACTCAACAAATC
GmVQ56	Glyma13g31180.1	F:AGCCCATGGACTCCATTTCCCCAAA<br>R:AGCTCTAGACTAAGAAGGAGGAGCGGAAGA
GmVQ57	Glyma13g35130.1	F:AGCCCATGGAAATCAGCTCAAGGCT<br>R:AGCTCTAGATCAAGAAGGTGATTGTGATGAGA
GmVQ58	Glyma14g00570.1	F:AGCCTCGAGATGACTGCTACCATGTCTGG<br>R:AGCTCTAGATCAAGAAGAAGTTGTTGGGAAGA
GmVQ59	Glyma14g29580.2	F:AGCCTCGAGATGATTAACAAGGACTCCCACAA<br>R:AGCTCTAGATCAAGGTGAAACAAAGCTTGAA
GmVQ60	Glyma14g34681.1	F:AGCCTCGAGATGGATTCTGGCAACAGTGG<br>R:AGCTCTAGAGTGGGGAAGGACGCAAAG
GmVQ61	Glyma15g08160.1	F:AGCCCATGGACTCCATTTCCCCAA<br>R:AGCTCTAGATTAAGAAGGAGGAGCGGAAG

GmVQ62	Glyma15g16990.1	F:AGCCTCGAGATGAGGGATATGACCATGGC<br>R:AGCGGATCCCTATAATTCACTCTTATAAAAGGTATCATTGG
GmVQ63	Glyma15g37230.1	F:AGCCTCGAGATGAAGAACAAAAGGGCAAGC<br>R:AGCTCTAGACTATGACATGTCAAAACTCATCAAATC
GmVQ64	Glyma15g40000.1	F:AGCCTCGAGATGTCTACCAGTGGATGCACC<br>R:AGCTCTAGATTAATCAGCCCAAAGGTCGT
GmVQ65	Glyma15g42280.2	F:AGCCTCGAGATGACAAAGCTACACACTTGTTATCC<br>R:AGCTCTAGATTAATTTTGATTCTCTGAAGGCACA
GmVQ66	Glyma17g17210.1	F:AGCCCATGGAAGCCTATTCTGGTTCTT<br>R:AGCTCTAGAGAACCTTCCCCGGTTCCAA
GmVQ67	Glyma18g02140.1	F:AGCCTCGAGATGGATTCGGGCAACAGT<br>R:AGCACTAGTTAATCAGAAGAACAATTAATCCAGG
GmVQ70	Glyma19g31080.1	F:AGCCCATGGATACCATTCGTACCACC<br>R:AGCTCTAGATCACCAGTGATTAAGATGAGGGGA
GmVQ71	Glyma19g38980.1	F:AGCCTCGAGATGAATTCCCAAGCAGCAA<br>R:AGCTCTAGATTATACAAAATCTACAAAGTGCATT
GmVQ72	Glyma19g43590.1	F:AGCCTCGAGATGCAAAAAACTCCAAAGAAAAC<br>R:AGCTCTAGATCACGGCAAAACAGAACG
GmVQ73	Glyma20g15230.1	F:AGCCTCGAGATGACCCAAACCATGTCAGG<br>R:AGCTCTAGATCAAGAAGAAGAAGAAGTGTTCG
GmVQ74	Glyma20g25070.2	F:AGCCTCGAGATCAATGGTCCACGTCCTTC<br>R:AGCTCTAGATCAATATTCAGGCAACTCTTTCA

Supplemental Table 6. Primers for generating VQ gene mutants.

Gene name	Gene identifier	Primers[a]
GmVQ7	Glyma03g28360.1	F:CCAACCCCATGGTGATCAAGACCA<br>R:TGGTCTTGATCACCATGGGGTTGG
GmVQ16	Glyma05g26340.1	F:CCAATTACCCTTATCAAAGCCAACA<br>R:TGTTGGCTTTGATAAGGGTAATTGG
GmVQ27	Glyma07g03240.1	F:AGCGAATTCATGGCTGCTTCCAACTCGACCCTCCAC<br>TCACCCACCACTCCCAACACCACCATCGT<br>R:AGCTCTAGATTAATTAGGAATTGATGGTGAATTG
GmVQ35	Glyma08g15620.1	F:CCGTGTCTCCGCTAGTCCTCCAC<br>R:GTGGAGGACTAGCGGAGACACGG
GmVQ44	Glyma09g17151.1	F:ATATTTGCACCCCTGATCATAAAAACGGA<br>R:TCCGTTTTTATGATCAGGGGTGCAAATAT
GmVQ47	Glyma11g04970.1	F:ACCGATCAGAGTGTTGAAGGTGGACGCT<br>R:AGCGTCCACCTTCAACACTCTGATCGGT

[a]The mutated codons are shown in red.
